# Accurate long-read sequencing identified *GBA1* as major risk factor in the Luxembourgish Parkinson’s study

**DOI:** 10.1038/s41531-023-00595-w

**Published:** 2023-11-23

**Authors:** Sinthuja Pachchek, Zied Landoulsi, Lukas Pavelka, Claudia Schulte, Elena Buena-Atienza, Caspar Gross, Ann-Kathrin Hauser, Dheeraj Reddy Bobbili, Nicolas Casadei, Patrick May, Rejko Krüger, on behalf of the NCER-PD Consortium, Geeta Acharya, Gloria Aguayo, Myriam Alexandre, Muhammad Ali, Wim Ammerlann, Giuseppe Arena, Rudi Balling, Michele Bassis, Roxane Batutu, Katy Beaumont, Regina Becker, Camille Bellora, Guy Berchem, Daniela Berg, Alexandre Bisdorff, Ibrahim Boussaad, David Bouvier, Kathrin Brockmann, Jessica Calmes, Lorieza Castillo, Gessica Contesotto, Nancy De Bremaeker, Nico Diederich, Rene Dondelinger, Nancy E. Ramia, Daniela Esteves, Guy Fagherazzi, Jean-Yves Ferrand, Katrin Frauenknecht, Manon Gantenbein, Thomas Gasser, Piotr Gawron, Soumyabrata Ghosh, Marijus Giraitis, Enrico Glaab, Martine Goergen, Elisa Gómez De Lope, Jérôme Graas, Mariella Graziano, Valentin Groues, Anne Grünewald, Wei Gu, Gaël Hammot, Anne-Marie Hanff, Linda Hansen, Michael Heneka, Estelle Henry, Sylvia Herbrink, Sascha Herzinger, Michael Heymann, Michele Hu, Alexander Hundt, Nadine Jacoby, Jacek Jaroslaw Lebioda, Yohan Jarosz, Sonja Jónsdóttir, Quentin Klopfenstein, Jochen Klucken, Rejko Krüger, Pauline Lambert, Roseline Lentz, Inga Liepelt, Robert Liszka, Laura Longhino, Victoria Lorentz, Paula Cristina Lupu, Tainá M. Marques, Clare Mackay, Walter Maetzler, Katrin Marcus, Guilherme Marques, Patricia Martins Conde, Deborah Mcintyre, Chouaib Mediouni, Francoise Meisch, Myriam Menster, Maura Minelli, Michel Mittelbronn, Brit Mollenhauer, Friedrich Mühlschlegel, Romain Nati, Ulf Nehrbass, Sarah Nickels, Beatrice Nicolai, Jean-Paul Nicolay, Fozia Noor, Marek Ostaszewski, Clarissa P. C. Gomes, Claire Pauly, Laure Pauly, Lukas Pavelka, Magali Perquin, Rosalina Ramos Lima, Armin Rauschenberger, Rajesh Rawal, Kirsten Roomp, Eduardo Rosales, Isabel Rosety, Estelle Sandt, Stefano Sapienza, Venkata Satagopam, Margaux Schmitt, Sabine Schmitz, Reinhard Schneider, Jens Schwamborn, Raquel Severino, Amir Sharify, Ekaterina Soboleva, Kate Sokolowska, Hermann Thien, Elodie Thiry, Rebecca Ting Jiin Loo, Christophe Trefois, Johanna Trouet, Olena Tsurkalenko, Michel Vaillant, Mesele Valenti, Gilles Van Cutsem, Carlos Vega, Liliana Vilas Boas, Maharshi Vyas, Richard Wade-Martins, Paul Wilmes, Evi Wollscheid-Lengeling, Gelani Zelimkhanov

**Affiliations:** 1 LCSB, Luxembourg Centre for Systems Biomedicine, University of Luxembourg, Esch-Sur-Alzette, Luxembourg; 2 Parkinson Research Clinic, Centre Hospitalier de Luxembourg (CHL), Luxembourg, Luxembourg; 3 Transversal Translational Medicine, Luxembourg Institute of Health (LIH), Strassen, Luxembourg; 4 Department of Neurodegeneration, Center of Neurology, Hertie Institute for Clinical Brain Research, German Center for Neurodegenerative Diseases, University of Tübingen, Tübingen, Germany; 5 Institute of Medical Genetics and Applied Genomics, University of Tübingen, Tübingen, Germany; 6 NGS Competence Center Tübingen (NCCT), University of Tübingen, Tübingen, Germany; 7 Luxembourg Institute of Health, Strassen, Luxembourg; 8 Centre Hospitalier de Luxembourg, Strassen, Luxembourg; 9 Center of Neurology and Hertie Institute for Clinical Brain Research, Department of Neurodegenerative Diseases, University Hospital Tübingen, Tübingen, Germany; 10 Centre Hospitalier Emile Mayrisch, Esch-sur-Alzette, Luxembourg; 11 Laboratoire National de Santé, Dudelange, Luxembourg; 12 Association of Physiotherapists in Parkinson’s Disease Europe, Esch-sur-Alzette, Luxembourg; 13 Faculty of Science, Technology and Medicine, University of Luxembourg, Esch-sur-Alzette, Luxembourg; 14 Department of Epidemiology, CAPHRI School for Public Health and Primary Care, Maastricht University Medical Centre+, Maastricht, the Netherlands; 15 Centre Hospitalier du Nord, Ettelbrück, Luxembourg; 16 Oxford Parkinson’s Disease Centre, Nuffield Department of Clinical Neurosciences, University of Oxford, Oxford, UK; 17 Private practice, Ettelbruck, Luxembourg; 18 Parkinson Luxembourg Association, Leudelange, Luxembourg; 19 Westpfalz-Klinikum GmbH, Kaiserslautern, Germany; 20 Oxford Centre for Human Brain Activity, Wellcome Centre for Integrative Neuroimaging, Department of Psychiatry, University of Oxford, Oxford, UK; 21 Department of Neurology, University Medical Center Schleswig-Holstein, Kiel, Germany; 22 Ruhr-University of Bochum, Bochum, Germany; 23 Paracelsus-Elena-Klinik, Kassel, Germany; 24 Private practice, Luxembourg, Luxembourg; 25 Oxford Parkinson’s Disease Centre, Department of Physiology, Anatomy and Genetics, University of Oxford, South Parks Road, Oxford, UK

**Keywords:** Parkinson's disease, Parkinson's disease

## Abstract

Heterozygous variants in the glucocerebrosidase *GBA1* gene are an increasingly recognized risk factor for Parkinson’s disease (PD). Due to the *GBAP1* pseudogene, which shares 96% sequence homology with the *GBA1* coding region, accurate variant calling by array-based or short-read sequencing methods remains a major challenge in understanding the genetic landscape of *GBA1*-associated PD. We analyzed 660 patients with PD, 100 patients with Parkinsonism and 808 healthy controls from the Luxembourg Parkinson’s study, sequenced using amplicon-based long-read DNA sequencing technology. We found that 12.1% (77/637) of PD patients carried *GBA1* variants, with 10.5% (67/637) of them carrying known pathogenic variants (including severe, mild, risk variants). In comparison, 5% (34/675) of the healthy controls carried *GBA1* variants, and among them, 4.3% (29/675) were identified as pathogenic variant carriers. We found four *GBA1* variants in patients with atypical parkinsonism. Pathogenic *GBA1* variants were 2.6-fold more frequently observed in PD patients compared to controls (OR = 2.6; CI = [1.6,4.1]). Three novel variants of unknown significance (VUS) were identified. Using a structure-based approach, we defined a potential risk prediction method for VUS. This study describes the full landscape of *GBA1*-related parkinsonism in Luxembourg, showing a high prevalence of *GBA1* variants as the major genetic risk for PD. Although the long-read DNA sequencing technique used in our study may be limited in its effectiveness to detect potential structural variants, our approach provides an important advancement for highly accurate *GBA1* variant calling, which is essential for providing access to emerging causative therapies for *GBA1* carriers.

## Introduction

Heterozygous variants in the glucocerebrosidase (*GBA1*) gene, which encodes the enzyme β-glucocerebrosidase (GCase), are increasingly recognized as the most common genetic risk factor for the development of Parkinson’s disease (PD). Homozygous variants in *GBA1* are causative for the most frequent autosomal-recessive lysosomal storage disorder, Gaucher disease (GD)^1^. GD is characterized by a deficiency of the enzyme GCase which is required to hydrolyze the β-glucosyl linkage of glucosylceramide lipide in lysosomes to form glucose and ceramide^2^.

Accurate variant calling in the *GBA1* gene is challenging due to the presence of the highly homogeneous untranslated pseudogene called *GBAP1*, which is located 16 kilobases downstream^3^, and shares 96% sequence homology within the coding region^4^. In addition, recombination and structural chromosomal variation within and around the *GBA1* locus further complicate the analysis^5^. Complex alleles, which include several single nucleotide variants, are derived from recombination between the functional *GBA1* gene and the *GBAP1* pseudogene^6^. RecNciI is the most common recombinant allele, including the amino acid changes p.L483P and p.A495P, and the synonymous variant p.V499V^6^.

Our study aimed to accurately assess all rare coding variants in the *GBA1* gene in all participants of the Luxembourg Parkinson’s study^7^, a case and control cohort including patients with PD and atypical parkinsonism. To assess the accuracy of the targeted *GBA1* DNA sequencing method using the Pacific Biosciences (PacBio)^8^ technology, which targets only the *GBA1* gene without sequencing the *GBAP1* pseudogene, we compared this method with genotyping using the NeuroChip array^9^ and short-read whole genome sequencing (WGS) data using Sanger sequencing as the gold standard for validation. We identified several types of pathogenic *GBA1* variants (severe, mild, and risk) and further characterized genotype–phenotype associations to better understand the influence of each variant type and their effect on disease severity.

## Results

### Demographic and clinical characteristics

**Fig. 1:**
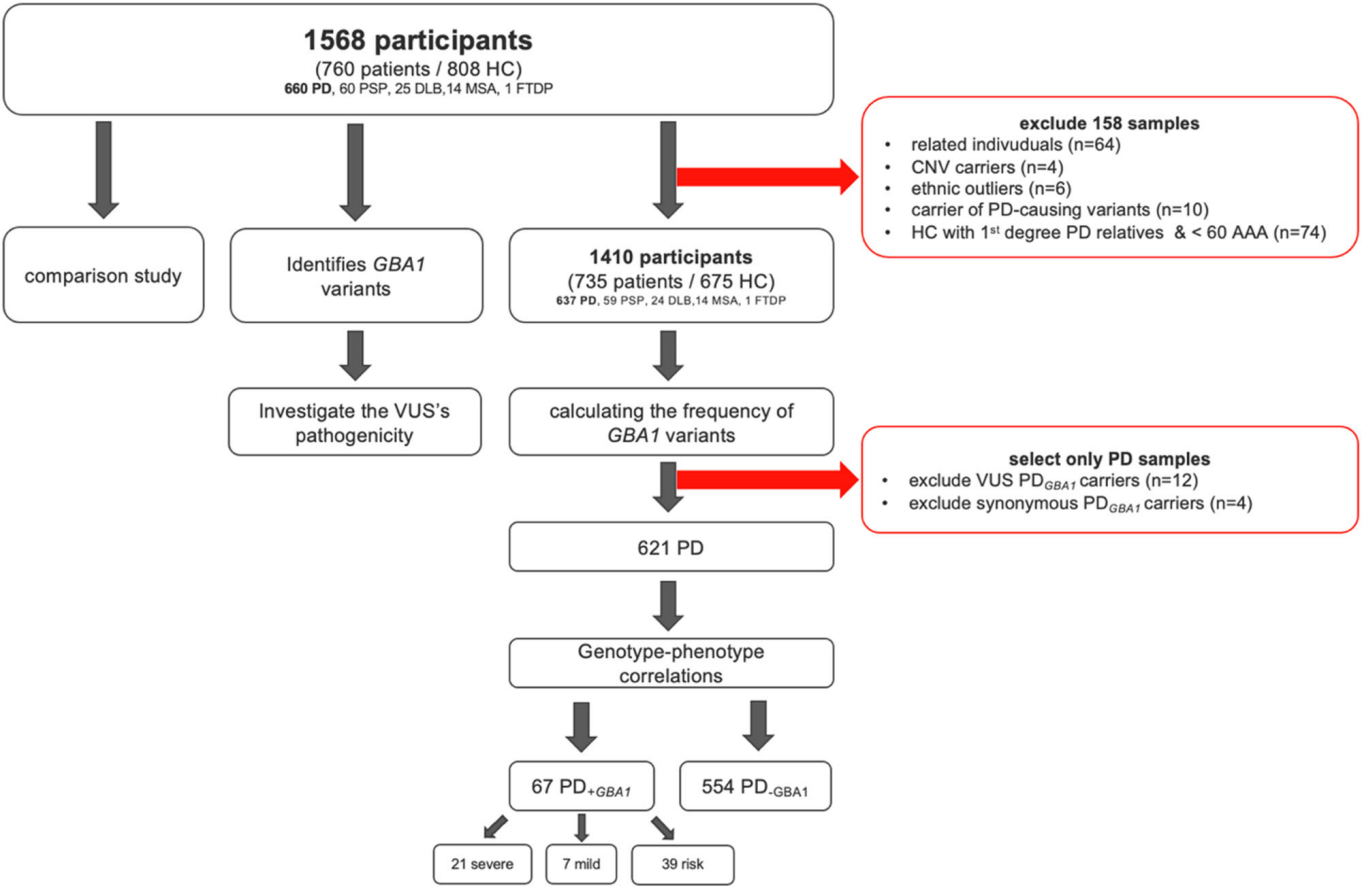
Description of the study dataset and methodology. HC Healthy controls, PD Parkinson’s Disease and Parkinson’s Disease with Dementia, PSP Progressive Supranuclear Palsy, DLB Dementia with Lewy Body, MSA Multiple System Atrophy, FTDP Fronto-temporal dementia with parkinsonism, *GBA1* glucocerebrosidase gene, VUS Variants of unknown significance, PD_+*GBA1*_ PD patients with *GBA1* pathogenic variant, PD_-*GBA1*_ PD patients without *GBA1* pathogenic variant, CNV copy number variants, AAA age at assessment.

A total of 760 patients (660 PD patients (*n*_PD_) and 100 patients with other forms of parkinsonism (*n*_park_)) and 808 healthy controls (*n*_HC_) from the Luxembourg Parkinson’s study (Fig. 1) were genotyped using NeuroChip and screened for *GBA1* variants using targeted PacBio DNA sequencing method, while a subset of 72 patients was screened with WGS. Among the patients, 66.4% (*n* = 499) were male with a mean age at disease onset (AAO) of 63 ± 11.5 years (Supplementary Table 1). The control group consisted of 52.7% (*n* = 426) males with a mean age at assessment (AAA) of 59.3 ± 12.2 years. Due to their above 30-fold coverage provided by the long-read DNA sequencing, all samples were selected after successfully passing the MultiQC step (Supplementary Table 9). To ensure ethnic homogeneity and exclude other genetic factors that may bias the assessment of the genetic contribution of *GBA1* to PD in the Luxembourgish population, we excluded carriers of mutations in other PD-causing genes (point mutations: *n* = 10, *n*_PD_ = 8,*n*_HC_ = 2; CNV: *n*_PD_ = 4) in PD-associated genes (no CNVs in *GBA1* were detected), first-degree family members (*n* = 64, *n*_PD_ = 8, *n*_park_ = 2, *n*_HC_ = 54), younger HC (<60 AAA) with first-degree relatives having PD (*n*_HC _= 74), and individuals of non-European descent (*n* = 6) from the cohort. The final cohort consisted of 735 patients (*n*_PD_=637, *n*_park_ = 98) and 675 HC with a mean AAO among the patients of 63.2 ± 11.3 years, whereas the mean AAA for HC was 61 ± 11.5 years. Based on Neurochip and WGS data, none of the *GBA1* carriers carried pathogenic variants in other PD-associated genes as defined by MDSGene^10^.

### Targeted PacBio DNA sequencing showed the highest specificity for detecting rare coding variants in *GBA1*

**Fig. 2:**
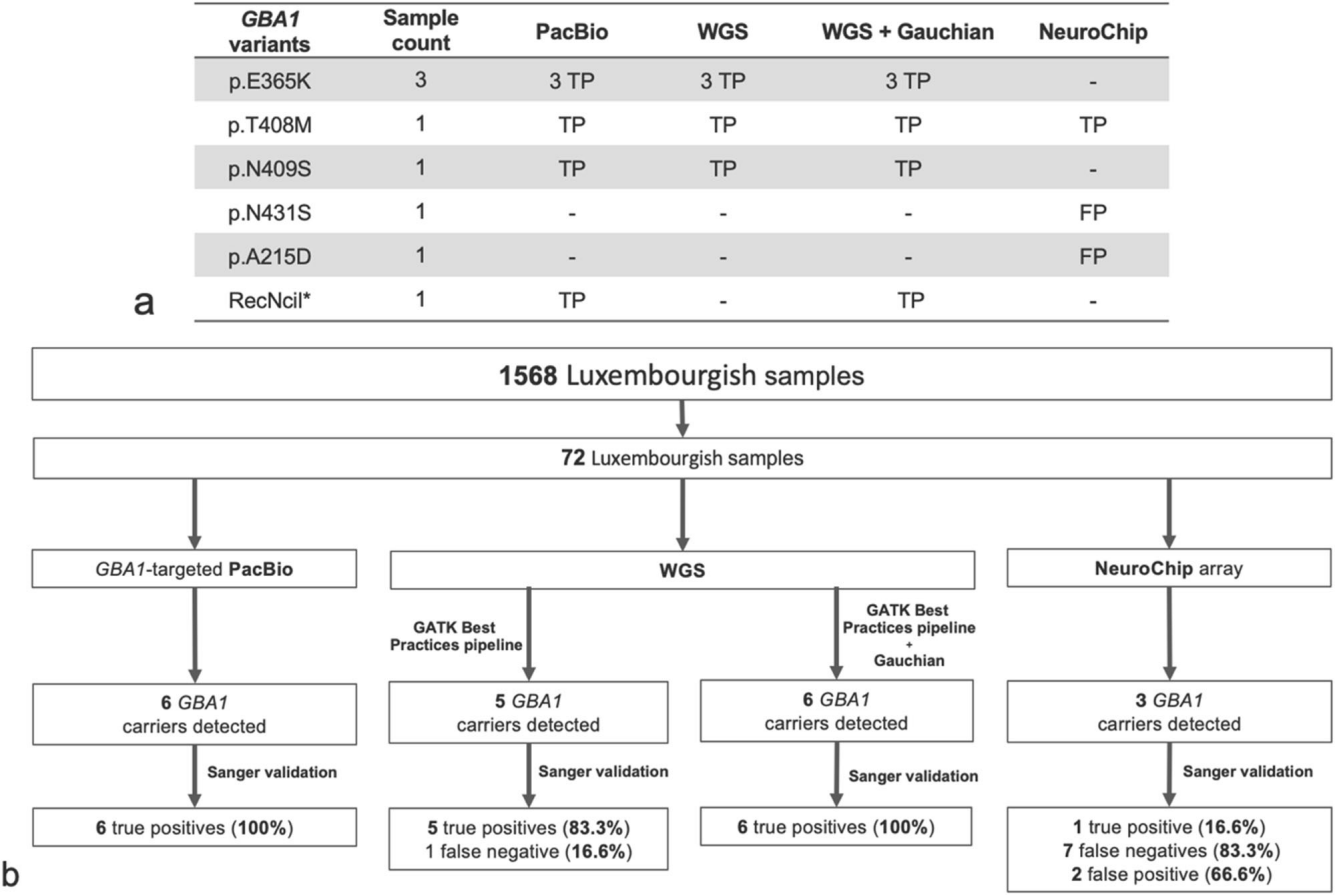
Comparison of variant calls from PacBio, WGS and NeuroChip genotyping data using 72 matched samples for the *GBA1* gene and validated by Sanger sequencing. **a** *RecNcil (p.L483P; p.A495P; p.V499V); Sanger sequencing results: TP, true positive; FP, false positive. Sample count gives the total number of samples carrying the variant found by each method. **b** Comparative study of *GBA1* variants detection by the *GBA1*-targeted PacBio DNA sequencing method and NeuroChip array methods in the Luxembourg Parkinson’s study. Due to overrepresented variants with the NeuroChip array, we applied for the detected variants a study-wide threshold of 1% in our cohort.

To measure the reliability of calling rare *GBA1* coding variants, we performed two types of comparison. Rare variants were here defined as variants with minor allele frequency (MAF) < 1% in the European population. We compared the results from the PacBio, WGS, and NeuroChip data for a subset of samples (*n* = 72). We then compared the PacBio and NeuroChip data as they both covered the majority of samples (*n* = 1568). We considered true positives to be the *GBA1* variants validated by Sanger sequencing. False-positive variants were those identified by the analysis method but not confirmed by Sanger sequencing. False-negative variants were not called by the analysis method but were later validated with Sanger sequencing (Supplementary Table 2). First, we evaluated 72 samples screened by all three methods (Fig. 2). Using the *GBA1*-targeted PacBio DNA sequencing method and WGS in combination with the Gauchian^11^ tool implemented in Dragen v4 (GBA caller option), we detected six individuals carrying *GBA1* variants (p.E365K (*n* = 3), p.T408M (*n* = 1), p.N409S (*n* = 1), RecNciI (*n* = 1)). The RecNil combines the three variants p.L483P, p.A495P, and p.V499V in one haplotype allele. All variants detected were confirmed by Sanger sequencing (true positive rate (TPR) of 100%). We did not identify any false positive variant calls. However, using the Dragen v.4 pipeline without the *GBA1* caller, relying only on the GATK best practices pipeline, the WGS method failed to detect the RecNciI recombinant allele in one individual (TPR of 83.3% (5/6)). Using Neurochip, we detected three potential *GBA1* variant carriers (p.T408M (*n* = 1), p.N431S (*n* = 1), p.A215D (*n* = 1), but only one variant (p.T408M) was subsequently confirmed by Sanger sequencing (TPR of 16.6% (1/6), resulting in a false discovery rate (FDR) of 66.6% (2/3).

**Fig. 3:**
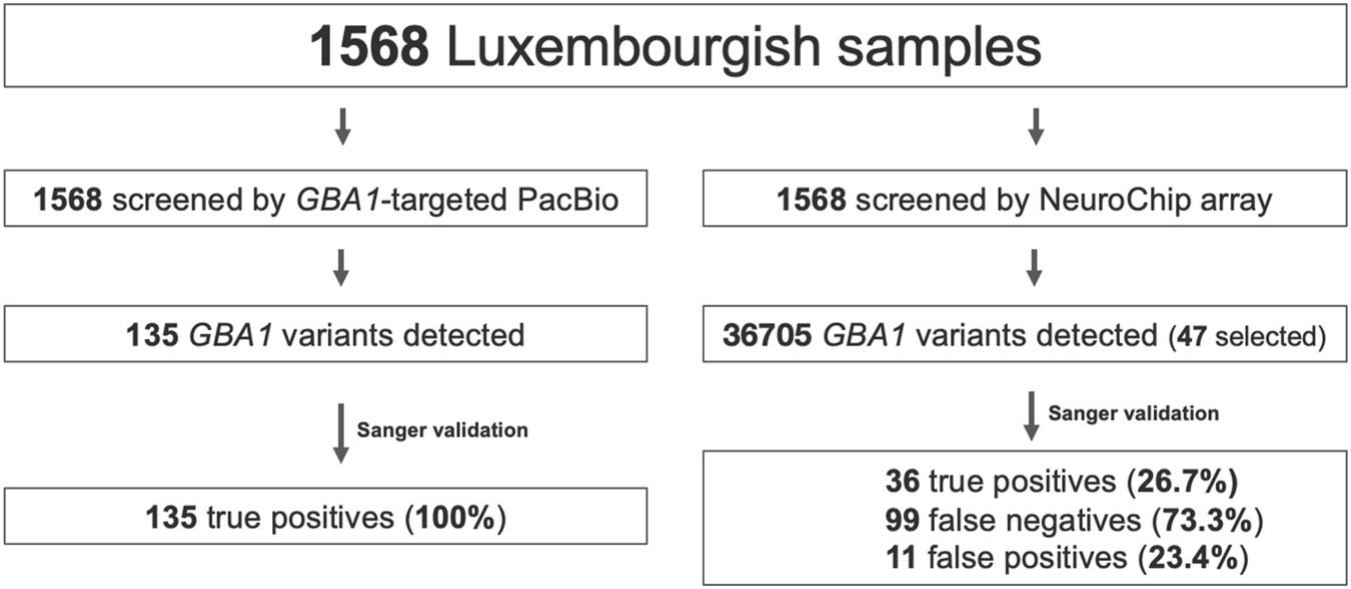
Comparative study of *GBA1* variants detection by the GBA1-targeted PacBio DNA sequencing and NeuroChip array methods in the Luxembourg Parkinson’s study. Due to overrepresented variants with the NeuroChip array, we applied for the detected variants a study-wide threshold of 1% in our cohort.

Next, we compared the results from 1568 samples screened with both, the *GBA1-targeted* PacBio DNA sequencing method and the NeuroChip array (Fig. 3). Using the GBA1-targeted PacBio DNA method, we detected 135 *GBA1* variants carriers, of which 100% were validated by Sanger sequencing. Using the NeuroChip array, we detected 47 potential *GBA1* variant carriers, among which only 36 were validated by Sanger sequencing (TPR of 26.7% (36/133), resulting in an FDR of 23.4% (11/47).

### Classification of *GBA1* variants

Of the 1568 individuals sequenced using the *GBA1*-targeted PacBio DNA sequencing method, we identified 135 carriers of at least one *GBA1* variant (Supplementary Tables 3, 4). Based on the classification of Höglinger et al.^12^, 25 were carriers of severe variants, 10 of mild variants, 72 of risk variants and 22 of VUS. The most common *GBA1* variants in PD patients were the risk variants p.E365K (*n* = 23; 3.5%) and p.T408M (n = 17; 2.6%).

*GBA1* variants were mostly heterozygous missenses, one patient carried a heterozygous stop-gain variant p.R398*(rs121908309), two PD patients carried a homozygous missense variant p.E365K/p.E365K(rs2230288). We identified two HC carrying a pathogenic *LRRK2* variant and being positive for *GBA1* variant (p.E365K_GBA_-p.R1441C_LRRK2_; p.K13R_GBA_-p.G2019S_LRRK2_). We also detected nine different synonymous variants in exonic regions (Supplementary Table 4). The variant p.T408T(rs138498426) is a splice site variant (located within 2 bp of the exon boundary) and is classified as VUS^12^. The remaining synonymous variants were not further analyzed. Additionally, we identified 69 variants in intronic and UTRs regions of *GBA1* (Supplementary Table 5) with unclear pathogenic relevance, 35 of which were rare with MAF < 1% in gnomAD for the Non-Finnish European population^10^.

We classified the following combinations of multiple variants per individual as severe based on the classification of the respective associated pathogenic variants (Table 1): p.N409S-p.L483P, p.K13R-p.L483P, p.F252I-p.T408M, p.Y61H-p.T408M.

**Table 1:** Distribution of GBA1 variants in the Luxembourg Parkinson’s study.

Subclassification	nucleotide - protein changes	Subjects	PD *n* = 660	Parkinsonism Patients *n* = 100	Healthy controls *n* = 808
Severe	c.115+1G>A	2	1		1
	p.P161S	2	2		
	p.G234W	1	1		
	p.G241R	2	2		
	p.H294Q	1			1
	p.R398X	1	1		
	p.G416S	1	1		
	p.L483P	6	5		1
	p.R502H	1	1		
	p.N409S; p.L483P	1	1		
	RecNciI*	5	4		1
	p.K13R; p.L483P	1	1		
	p.F252I; p.T408M	1	1		
Mild	p.N409S	10	7		3
Risk	p.E365K	42	21 + 2^a^	1 DLB + 2 PSP	16
	p.E365K (*LRRK2*: p.R1441C)*	1			1
	p.T408M	28	15	1 DLB	12
	p.Y61H; p.T408M	1	1		
VUS	p.K13R	4	2		2
	p.K13R (*LRRK2*: p.G2019S)*	1			1
	p.Y61H	1	1		
	p.R78C	2			2
	p.A97G (new VUS)	1			1
	p.L213P	1	1		
	p.A215D (new VUS)	1	1		
	p.E427K	2	1		1
	p.R434C (new VUS)	1	1		
	p.H529R	1	1		
	p.R534C	1	1		
	p.A495P; p.V499V	3	1		2
	p.T408T	3	2		1

All variants were identified in the heterozygous state except in two individuals for p.E365K *RecNcil (p.L483P; p.A495P; p.V499V); *LRRK2 mutation in brackets.

*GBA1* glucocerebrosidase gene, *PD* Parkinson’s Disease and Parkinson’s Disease with Dementia, *PSP* Progressive Supranuclear Palsy, *DLB* Dementia with Lewy Body, *VUS* Variants of unknown significance.

^a^Homozygous state.

### *GBA1* variant frequency

To calculate the *GBA1* frequency in our study, we considered the individuals remaining after the exclusion step (735 patients and 675 HC). We detected 12.1% (*n* = 77) *GBA1* variant carriers among 637 PD patients and 5% (34/675) in HC individuals. We found a frequency of 10.5% (67/637) of pathogenic variants in PD patients (severe, mild, risk) and 4.3% (29/675) in controls (Table 2). Four patients with parkinsonism had *GBA1* variants. Carriers of severe *GBA1* variants (*n* = 21; 3.2%; OR = 11.4; 95% CI = [2.6, 49]; *p* = 0.0010) have a high risk of developing PD as defined by the indicated OR.

**Table 2:** Frequency of GBA1 variants in the Luxembourg Parkinson’s study.

Diagnosis	Sub-classification of *GBA1* variants	Subjects	All GBA1-Carrier *n* (%)	Pathogenic *GBA1*-Carrier *n* (%)	OR (95%CI)	*p* values
PD		637	**77 (12.1%)**	**67 (10.5%)**	**2.6 (1.6–4.1)**	0.0001^*^
	**severe**			**21 (3.2%)**	**11.4 (2.6–49)**	0.0010^*^
	mild			7 (1.1%)	3.7 (0.7 to 18)	0.1008
	**risk**			**39 (6%)**	**1.6 (1 to 2.8)**	0.0537^*^
PSP	risk	59		2 (3.4%)	0.9 (0.2–3.9)	0.8941
DLB	risk	24		2 (8.3%)	2.2 (0.5–10)	0.2908
Healthy controls		675	34 (5%)	29 (4.3%)	-	-
	severe			2	-	-
	mild			2	-	-
	risk			25	-	-

Subject numbers result from excluding the first-degree family members interrelated in the cohort, the healthy controls of young age of assessment (<60 AAA) with first-degree PD relatives, the CNV carriers, carrier of PD-causing variants (except GBA1) and the ethnic outliers.

*GBA1* glucocerebrosidase gene, *PD* Parkinson’s Disease and Parkinson’s Disease with Dementia, *PSP* progressive supranuclear palsy, *DLB* Dementia with Lewy Body.

ORs are given with the 95% CI; Statistically significant results are highlighted in bold with a * sign (*p* value < 0.05).

### Genotype–phenotype associations in *GBA1*-PD patients

We characterized the clinical phenotype of severe (*n* = 21), mild (*n* = 7) and risk (*n* = 39) *GBA1* carriers and non-carriers (*n* = 554) only in unrelated PD patients excluding carriers with only one synonymous or VUS variant in individuals remaining after the filtering step. The AAO was similar between *GBA1* carriers (61.6 ± 11.5) and non-carriers (62.6 ± 11.6). Severe PD_*GBA1*_ variant carriers showed a trend towards younger AAO compared to mild and risk (severe: 58.6 ± 13.1 vs mild: 65.4 ± 17 vs risk: 62.5 ± 9.3 years; *p* = 0.29) (Table 3), with a significant risk to develop early onset PD (OR = 4.02; *p* = 0.0098). In contrast to non-carriers, we also observed that carriers of pathogenic variants have a strong family history of PD (OR = 0.74; *p* = 0.0401).

**Table 3:** Demographic data for the PD patients in the Luxembourg Parkinson’s study separated by *GBA1* variant status.

Features	All pathogenic variants (*n* = 67)	Severe (*n* = 21)	Mild (*n* = 7)	Risk (*n* = 39)	Non carriers (*n* = 554)
AAA, mean (SD)	66.5 (±10.2) [OR = 0.31; *p* = 0.3977]	65.1 (±10.2) [OR = 0.08; *p* = 0.292]	67.1 (±15.6) [OR = 0.59; *p* = 0.8959]	67.1 (±9.2) [OR = 0.57; *p* = 0.7512]	67.6 (±10.7)
Sex, Male *n* (%)	40 (59.7%) [OR = 0.71; *p* = 0.1912]	13 (61.9%) [OR = 0.78; *p* = 0.5795]	5 (71.4%) [OR = 1.19; *p* = 0.8336]	22 (56.4%) [OR = 0.62; *p* = 0.151]	375 (67.7%)
AAO, mean (SD)	61.6 (±11.5) [OR = 0.35; *p* = 0.484]	58.6 (±13.1) [OR = 0.02; *p* = 0.1158]	65.4 (±17.0) [OR = 16.16; *p* = 0.5308]	62.5 (±9.3) [OR = 0.9; *p* = 0.9548]	62.6 (±11.6)
AAO < 45, *N* (%)	8 (11.9%) [OR = 1.74; *p* = 0.1767]	**5 (23.8%) [OR** **=** **4.02;** ***p*** **=** 0.0098*]	2 (28.6%) [OR = 5.14; *p* = 0.0549]	1 (2.6%) [OR = 0.34; *p* = 0.2907]	40 (7.2%)
Disease Duration, mean (SD)	4.7 (±4.8) [OR = 0.79; *p* = 0.7303]	6.4 (±4.7) [OR = 4.07; *p* = 0.2238]	1.7 (±1.4) [OR = 0.04; *p* = 0.0981]	4.4 (±4.9) [OR = 0.57; *p* = 0.5103]	5.0 (±5.2)
Family History, *N* (%)	**25 (37.3%) [OR** **=** **1.74;** ***p*** **=** 0.0401*]	8 (38.1%) [OR = 1.8; *p* = 0.2001]	2 (28.6% [OR = 1.17; *p* = 0.8508]	15 (38.5%) [OR = 1.83; *p* = 0.0782]	141 (25.5%)

Subject numbers result from excluding the first-degree family members interrelated in the cohort, the healthy controls with the young age of assessment (<60 AAA) or with first-degree PD relatives, the CNVs carriers, carrier of PD-causing variants (except *GBA1*), the ethnic outliers, synonymous and VUS variants carriers. Data are given as mean (SD) or *N* (%). Statistically significant results are highlighted in bold with a * sign (*p* value < 0.05).

*AAA* age at assessment in years, *AAO* Age at onset in years.

We compared clinical features between PD patients carrying pathogenic *GBA1* variants and PD patients without *GBA1* variants (Supplementary Table 6). We found that in carriers the sense of smell was strongly impaired (uncorrected *p* = 0.0210) and a higher rate of hallucinations (uncorrected *p* = 0.0415). Next, we compared patients carrying variants from each category (severe, mild or risk) separately with PD patients without *GBA1* variants (Table 4). Carriers of severe *GBA1* variants showed more severe non-motor symptoms when compared to non-*GBA1* carriers, such as MDS-UPDRS Part I (uncorrected *p* = 0.0074) and hallucinations (uncorrected *p* = 0.0099), and also an impaired sense of smell as assessed by Sniffin’ Stick test (uncorrected *p* = 0.0405). To show the deleterious impact of the severe variants, we compared carriers of severe variants with patients carrying either mild or risk *GBA1* variants (Table 5). We observed that severe variants carriers have more severe gait disorder (uncorrected *p* = 0.0188) and depression (uncorrected *p* = 0.0074) and worse MDS-UPDRS Part I (uncorrected *p* = 0.0019) and PDQ-39 (uncorrected *p* = 0.0422). For all clinical features, there were no significant associations after the correction for multiple comparisons using FDR adjustment.

**Table 4:** Clinical characteristics of PD classified by *GBA1* variant status.

		PD *GBA1* carrier															PD GBA1 non-carrier
		SEVERE					MILD					RISK					*N* = 554
Type of data	Clinical characteristics and scales	PD_GBA1_ (*n* = 21)	Missing values (%)	*β* (95%)	*p* value	adj *p* value	PD_GBA1_ (*n* = 7)	missing values (%)	*β* (95%)	*p* value	adj *p* value	PD_GBA1_ (*n* = 39)	Missing values (%)	*β* (95%)	*p* value	adj *p* value	
**Motor symptoms/scales**	**H&Y, mean (SD)**	2.4 ( ± 0.8)	3 (0.5%)	0.19 (−0.11 to 0.48)	0.2231	0.5354	2.0 ( ± 0.6)	3 (0.5%)	0.0 (−0.50 to 0.51)	0.9917	0.9992	2.2 ( ± 0.8)	3 (0.5%)	0.07 (−0.15 to 0.29)	0.5418	0.9471	2.2 ( ± 0.8)
	**MDS-UPDRS II, mean (SD)**	12.6 ( ± 4.4)	13 (2.3%)	0.49 (−2.75 to 3.72)	0.768	0.9678	10.1 ( ± 6.4)	13 (2.3%)	0.92 (−4.67 to 6.51)	0.7476	0.9992	11.1 ( ± 8.6)	13 (2.2%)	0.17 (−2.26 to 2.60)	0.8924	0.9748	11.4 ( ± 8.3)
	**MDS-UPDRS III, mean (SD)**	34.8 ( ± 15.7)	14 (2.4%)	−0.15 (−7.08 to 6.78)	0.966	0.9688	26.2 ( ± 9.7)	13 (2.3%)	−4.62 (−16.79 to 7.54)	0.4564	0.9992	33.3 ( ± 17.7)	12 (2.0%)	−0.37 (−5.30 to 4.57)	0.8846	0.9748	34.6 ( ± 16.2)
	**MDS-UPDRS IV, mean (SD)**	3.0 ( ± 4.5)	7 (1.2%)	0.87 (−0.46 to 2.20)	0.1991	0.512	0.1 ( ± 0.4)	7 (1.2%)	−0.63 (−2.86 to 1.60)	0.5816	0.9992	1.2 ( ± 2.3)	7 (1.2%)	−0.42 (−1.38 to 0.54)	0.395	0.8145	1.6 ( ± 3.3)
	**Dyskinesias,** ***n*** **(%)**	5 (23.8%)	0	0.67 (−0.48 to 1.82)	0.2535	0.5368	0	-	-	-	-	4 (10.3%)	0	0.01 (−1.13 to 1.15)	0.9853	0.9928	64 (11.6%)
	**Falls,** ***n*** **(%)**	5 (23.8%)	0	0.46 (−0.62 to 1.53)	0.4054	0.6081	0	-	-	-	-	7 (17.9%)	0	0.17 (−0.74 to 1.08)	0.7103	0.9471	93 (16.8%)
	**Gait Disorder,** ***n*** **(%)**	16 (76.2%)	0	0.94 (−0.09 to 1.97)	0.075	0.512	3 (42.9%)	0	−0.24 (−1.76 to 1.29)	0.7627	0.9992	18 (46.2%)	0	−0.29 (−0.97 to 0.38)	0.3953	0.8145	307 (55.4%)
	**FOG,** ***n*** **(%)**	8 (38.1%)	0	0.66 (−0.33 to 1.65)	0.1932	0.512	0	-	-	-	-	7 (17.9%)	0	−0.19 (−1.12 to 0.74)	0.69	0.9471	123 (22.2%)
	**Restless leg syndrome,** ***n*** **(%)**	2 (9.5%)	0	0.03 (−1.47 to 1.53)	0.9688	0.9688	2 (28.6%)	0	1.61 (−0.09 to 3.3)	0.0632	0.8508	6 (15.4%)	0	0.7 (−0.23 to 1.63)	0.141	0.8145	46 (8.3%)
	**Motor fluctuation,** ***n*** **(%)**	5 (23.8%)	0	0.1 (−1.05 to 1.26)	0.8626	0.9678	0	-	-	-	-	5 (12.8%)	0	−0.22 (−1.25 to 0.82)	0.6834	0.9471	93 (16.8%)
**Non-motor symptoms/scales**	**BDI, mean (SD)**	12.4 ( ± 5.7)	28 (4.9%)	2.09 (−0.91 to 5.1)	0.1721	0.512	8.0 ( ± 3.5)	29 (5.2%)	−1.13 (−6.69 to 4.42)	0.6894	0.9992	8.0 ( ± 5.7)	29 (4.9%)	−1.87 (−4.12 to 0.38)	0.1045	0.8145	9.9 ( ± 7.1)
	**MDS-UPDRS Part I, mean (SD)**	**15.0 (** ± **6.5)**	**15 (2.6%)**	**3.99 (1.06 to 6.92)**	0.0074*	**0.1782**	8.6 ( ± 2.4)	15 (2.7%)	−0.76 (−5.74 to 4.22)	0.7651	0.9992	9.5 ( ± 6.8)	15 (2.5%)	−0.9 (−3.06 to 1.27)	0.417	0.8145	**10.5 (** ± **7.0)**
	**PDSS, mean (SD)**	98.3 ( ± 20.9)	44 (7.7%)	−4.62 (−15.31 to 6.08)	0.3978	0.6081	110.9 ( ± 13.4)	43 (7.7%)	2.24 (−15.58 to 20.05)	0.8055	0.9992	104.0 ( ± 24.9)	45 (7.6%)	−1.54 (−9.52 to 6.45)	0.7063	0.9471	105.0 ( ± 24.6)
	**SCOPA−AUT, mean (SD)**	17.1 ( ± 8.0)	32 (5.6%)	1.75 (−1.66 to 5.17)	0.3149	0.5967	13.1 ( ± 7.1)	31 (5.5%)	−0.19 (−5.89 to 5.50)	0.9469	0.9992	14.1 ( ± 7.8)	32 (5.4%)	−0.61 (−3.13 to 1.91)	0.6369	0.9471	14.9 ( ± 8.1)
	**Sniffin’s stick test, mean (SD)**	**6.4 (** ± **3.6)**	**7 (1.2%)**	**−1.56 (−3.05 to −0.06)**	0.0405*	**0.486**	6.6 ( ± 2.1)	7 (1.2%)	−1.56 (−4.10 to 0.97)	0.2281	0.9992	7.4 ( ± 4.0)	8 (1.3%)	−0.65 (−1.78 to 0.48)	0.2637	0.8145	**7.8 (** ± **3.6)**
	**SAS, mean (SD)**	15.8 ( ± 5.2)	33 (5.7%)	2.03 (−0.47 to 4.52)	0.1115	0.512	12.6 ( ± 3.8)	32 (5.7%)	−1.48 (−5.66 to 2.69)	0.4866	0.9992	13.1 ( ± 6.3)	34 (5.7%)	−0.76 (−2.64 to 1.12)	0.4299	0.8145	14.1 ( ± 5.7)
	**MoCA, mean (SD)**	24.0 ( ± 4.7)	12 (2.1%)	−0.78 (−2.58 to 1.02)	0.3947	0.6081	25.6 ( ± 3.7)	12 (2.1%)	0.87 (−2.17 to 3.91)	0.5757	0.9992	24.9 ( ± 4.0)	14 (2.4%)	0.14 (−1.22 to 1.49)	0.8443	0.9748	24.4 ( ± 4.5)
	**Constipation,** ***n*** **(%)**	10 (47.6%)	0	0.06 (−0.83 to 0.94)	0.903	0.9678	5 (71.4%)	0	1.39 (−0.27 to 3.05)	0.1002	0.9018	14 (35.9%)	0	−0.32 (−1.01 to 0.37)	0.3666	0.8145	246 (44.4%)
	**Dysphagia,** ***n*** **(%)**	4 (19.0%)	0	−0.48 (−1.59 to 0.64)	0.4015	0.6081	1 (14.3%)	0	−0.52 (−2.66 to 1.61)	0.6295	0.9992	10 (25.6%)	0	−0.0 (−0.75 to 0.75)	0.9928	0.9928	145 (26.2%)
	**Insomnia,** ***n*** **(%)**	6 (28.6%)	0	−0.05 (−1.04 to 0.93)	0.914	0.9678	4 (57.1%)	0	1.44 (−0.09 to 2.97)	0.0658	0.8508	7 (17.9%)	0	−0.56 (−1.41 to 0.28)	0.1929	0.8145	151 (27.3%)
	**Orthostatism,** ***n*** **(%)**	5 (23.8%)	0	−0.36 (−1.39 to 0.67)	0.4922	0.7088	4 (57.1%)	0	1.4 (−0.12 to 2.91)	0.0709	0.8508	15 (38.5%)	0	0.44 (−0.24 to 1.12)	0.2021	0.8145	163 (29.4%)
	**Urinary incontinence,** ***n*** **(%)**	6 (28.6%)	0	−0.08 (−1.06 to 0.9)	0.8713	0.9678	2 (28.6%)	0	0.15 (−1.54 to 1.83)	0.865	0.9992	17 (43.6%)	0	0.65 (−0.03 to 1.32)	0.0615	0.8145	168 (30.3%)
	**Hallucinations,** ***n*** **(%)**	**8 (38.1%)**	**0**	**1.23 (0.29 to 2.16)**	0.0099*	**0.1782**	2 (28.6%)	0	1.23 (−0.45 to 2.9)	0.1504	0.9024	6 (15.4%)	0	0.09 (−0.83 to 1.02)	0.8444	0.9748	**83 (15.0%)**
	**Excessive daytime sleepiness,** ***n*** **(%)**	9 (42.9%)	0	0.49 (−0.4 to 1.39)	0.2812	0.5624	0	-	-	-	-	14 (35.9%)	0	0.33 (−0.37 to 1.03)	0.3534	0.8145	170 (30.7%)
	**ICD,** ***n*** **(%)**	2 (9.5%)	0	−0.23 (−1.76 to 1.3)	0.7682	0.9678	0	-	-	-	-	4 (10.3%)	0	0.22 (−0.88 to 1.32)	0.6925	0.9471	53 (9.6%)
	**Syncope,** ***n*** **(%)**	2 (9.5%)	0	0.93 (−0.62 to 2.48)	0.2401	0.5368	1 (14.3%)	0	1.74 (−0.49 to 3.98)	0.1257	0.9024	3 (7.7%)	0	0.6 (−0.67 to 1.86)	0.3575	0.8145	26 (4.7%)
	**RBDSQ,** ***n*** **(%)**	10 (47.6%)	42 (7.3%)	0.65 (−0.28 to 1.59)	0.1727	0.512	1 (14.3%)	42 (7.5%)	−0.49 (−2.66 to 1.68)	0.6606	0.9992	14 (35.9%)	43 (7.3%)	0.42 (−0.31 to 1.15)	0.2631	0.8145	165 (29.8%)
**Other clinical outcomes**	**LEDD (mg/day), mean (SD)**	690.5 ( ± 457.9)	19 (3.3%)	117.68 (−35.58 to 270.94)	0.1324	0.512	324.7 ( ± 224.7)	20 (3.6%)	−68.22 (−349.43 to 212.99)	0.6345	0.9992	496.6 ( ± 443.2)	20 (3.4%)	7.86 (−107.30 to 123.02)	0.8936	0.9748	513.5 ( ± 404.9)
	**PDQ-39, mean (SD)**	52.0 ( ± 26.3)	49 (8.5%)	9.46 (−1.34 to 20.27)	0.0863	0.512	26.1 ( ± 17.7)	49 (8.7%)	−6.81 (−25.20 to 11.59)	0.4686	0.9992	34.9 ( ± 27.2)	51 (8.6%)	−4.36 (−12.59 to 3.86)	0.2987	0.8145	39.3 ( ± 26.7)
	**DBS,** ***n*** **(%)**	3 (14.3%)	0	1.38 (−0.21 to 2.98)	0.0882	0.512	0					1 (2.6%)	0	−0.07 (−2.25 to 2.11)	0.9477	0.9928	24 (4.3%)
**Comorbidities**	**Diabetes,** ***n*** **(%)**	2 (9.5%)	0	0.18 (−1.34 to 1.7)	0.8198	0.9678	1 (14.3%)	0	0.14 (−2.08 to 2.35)	0.9029	0.9992	5 (12.8%)	0	0.46 (−0.54 to 1.46)	0.3704	0.8145	55 (9.9%)
	**Hypercholesterolemia,** ***n*** **(%)**	6 (28.6%)	0	−0.44 (−1.42 to 0.54)	0.3784	0.6081	2 (28.6%)	0	−0.65 (−2.34 to 1.04)	0.4533	0.9992	17 (43.6%)	0	0.15 (−0.52 to 0.81)	0.6682	0.9471	226 (40.8%)
	**Cardiovascular disease,** ***n*** **(%)**	1 (4.8%)	0	−1.53 (−3.59 to 0.52)	0.1436	0.512	1 (14.3%)	0	−0.64 (−2.87 to 1.58)	0.5708	0.9992	8 (20.5%)	0	0.13 (−0.71 to 0.96)	0.7648	0.9748	116 (20.9%)
	**Arterial hypertension,** ***n*** **(%)**	9 (42.9%)	0	0.1 (−0.82 to 1.02)	0.8312	0.9678	2 (28.6%)	0	−0.88 (−2.6 to 0.85)	0.32	0.9992	12 (30.8%)	0	−0.57 (−1.28 to 0.15)	0.1197	0.8145	248 (44.8%)
	**Traumatic Brain Injury,** ***n*** **(%)**	5 (23.8%)	0	0.15 (−0.88 to 1.18)	0.7772	0.9678	0	-	-	-	-	6 (15.4%)	0	−0.43 (−1.33 to 0.46)	0.3421	0.8145	122 (22.0%)

Comparison of each type (severe, mild, risk) of GBA1 variants and its association with clinical characterization. We used regression models (linear and logistic). Data are given as mean and standard deviation (SD) for continuous clinical outcomes and as percentages for binary clinical outcomes. Models adjusted for sex, age at assessment, and disease duration. Beta (β) regression coefficients are given with the 95% CI. Statistically significant results are highlighted in bold with a * sign (*p* value < 0.05).

*p value* unadjusted p-value, *adj p value* corrected for multiple comparisons using FDR adjustment, *AAO* age at onset, *H&Y* Hoehn & Yahr, *MDS-UPDRS* Movement Disorders Society - Unified Parkinson’s Disease Rating Scale, *FOG* freezing of gait, *BDI* Beck Depression Inventory, *PDSS* Parkinson’s Disease Sleep Scale, *SCOPA-AUT* Scales for Outcomes in Parkinson’s Disease-Autonomic questionnaire, *SAS* Starkstein apathy scale, *MoCA* Montreal Cognitive Assessment, *ICD* impulse control disorder, *RBDSQ* REM Sleep Behavior Disorder Screening Questionnaire, *LEDD* L-dopa equivalent daily dose (mg/day), *PDQ-39* Parkinson’s Disease quality of life Questionnaire, *DBS* Presence of treatment by Deep Brain Stimulation.

**Table 5:** The deleterious impact of severe *GBA1-*PD carriers in comparison with mild and risk and their clinical characteristics.

Type of data	Clinical characteristics and scales	PD_GBA1_ carrier		Missing values (%)	*β* (95%)	*p* value	adj *p* value
		**severe (*n*** = **21)**	**mild + risk (*N*** = **46)**				
Motor symptoms/scales	H&Y, mean (SD)	2.4 ( ± 0.8)	2.2 ( ± 0.8)	0	0.14 (−0.24 to 0.52)	0.4719	0.7549
	MDS-UPDRS II, mean (SD)	12.6 ( ± 4.4)	10.9 ( ± 8.2)	0	0.24 (−3.18 to 3.65)	0.8922	0.9706
	MDS-UPDRS III, mean (SD)	34.8 ( ± 15.7)	32.4 ( ± 16.9)	3 (4.5%)	0.65 (−7.69 to 8.98)	0.8793	0.9706
	MDS-UPDRS IV, mean (SD)	3.0 ( ± 4.5)	1.0 ( ± 2.2)	0	1.33 (−0.17 to 2.82)	0.082	0.574
	Dyskinesias, *n* (%)	5 (23.8%)	4 (8.7%)	0	0.65 (−1.09 to 2.39)	0.4621	0.7549
	Falls, *n* (%)	5 (23.8%)	7 (15.2%)	0	0.24 (−1.29 to 1.77)	0.7594	0.9601
	**Gait Disorder,** ***n*** **(%)**	**16 (76.2%)**	**21 (45.7%)**	**0**	**1.49 (0.25 to 2.73)**	0.0188*	**0.2193**
	FOG, *n* (%)	8 (38.1%)	7 (15.2%)	0	0.79 (−0.73 to 2.32)	0.3091	0.7549
	Restless leg syndrome, *n* (%)	2 (9.5%)	8 (17.4%)	0	−0.98 (−2.81 to 0.85)	0.2952	0.7549
	Motor fluctuation, *n* (%)	5 (23.8%)	5 (10.9%)	0	0.28 (−1.36 to 1.92)	0.7348	0.9601
Non-motor symptoms/scales	**BDI, mean (SD)**	**12.4 (** ± **5.7)**	**8.0 (** ± **5.4)**	**2 (3.0%)**	**4.03 (1.08 to 6.98)**	0.0074*	**0.1295**
	**MDS-UPDRS Part I, mean (SD)**	**15.0 (** ± **6.5)**	**9.3 (** ± **6.3)**	**0**	**4.91 (1.8 to 8.02)**	0.0019*	**0.0665**
	PDSS, mean (SD)	98.3 ( ± 20.9)	105.1 ( ± 23.5)	3 (4.5%)	−2.79 (−14.9 to 9.33)	0.6521	0.9129
	SCOPA-AUT, mean (SD)	17.1 ( ± 8.0)	13.9 ( ± 7.6)	2 (3.0%)	2.61 (−1.62 to 6.85)	0.2269	0.7542
	Sniffin’s stick test, mean (SD)	6.4 ( ± 3.6)	7.3 ( ± 3.7)	1 (1.5%)	−0.86 (−2.75 to 1.02)	0.3695	0.7549
	SAS, mean (SD)	15.8 ( ± 5.2)	13.0 ( ± 6.0)	3 (4.5%)	2.02 (−0.94 to 4.97)	0.1817	0.7542
	MoCA, mean (SD)	24.0 ( ± 4.7)	25.0 ( ± 3.9)	2 (3.0%)	−0.24 (−2.4 to 1.92)	0.8292	0.9706
	Constipation, *n* (%)	10 (47.6%)	19 (41.3%)	0	0.12 (−1.02 to 1.26)	0.8394	0.9706
	Dysphagia, *n* (%)	4 (19.0%)	11 (23.9%)	0	−0.44 (−1.83 to 0.95)	0.5348	0.8138
	Insomnia, *n* (%)	6 (28.6%)	11 (23.9%)	0	−0.02 (−1.27 to 1.23)	0.9767	0.9836
	Orthostatism, *n* (%)	5 (23.8%)	19 (41.3%)	0	−0.61 (−1.83 to 0.61)	0.327	0.7549
	Urinary incontinence, *n* (%)	6 (28.6%)	19 (41.3%)	0	−0.76 (−1.97 to 0.44)	0.2156	0.7542
	Hallucinations, *n* (%)	8 (38.1%)	8 (17.4%)	0	1.03 (−0.24 to 2.3)	0.1127	0.6485
	Excessive daytime sleepiness, *n* (%)	9 (42.9%)	14 (30.4%)	0	0.41 (−0.71 to 1.52)	0.4745	0.7549
	ICD, *n* (%)	2 (9.5%)	4 (8.7%)	0	−0.02 (−1.89 to 1.85)	0.9836	0.9836
	Syncope, *n* (%)	2 (9.5%)	4 (8.7%)	0	0.1 (−1.72 to 1.92)	0.9151	0.9706
	RBDSQ, ***n*** **(%)**	10 (47.6%)	15 (32.6%)	4 (6.0%)	0.48 (−0.68 to 1.64)	0.4197	0.7549
Other clinical outcomes	LEDD (mg/day), mean (SD)	690.5 ( ± 457.9)	473.1 ( ± 422.4)	2 (3.0%)	66.4 (−107.09 to 239.89)	0.4531	0.7549
	**PDQ-39, mean (SD)**	**52.0 (** ± **26.3)**	**33.5 (** ± **25.9)**	**2 (3.0%)**	**12.77 (0.45 to 25.09)**	0.0422*	**0.3692**
	DBS, *n* (%)	3 (14.3%)	1 (2.2%)	0	0.76 (−2.17 to 3.69)	0.6122	0.8928
Comorbidities	Diabetes, *n* (%)	2 (9.5%)	6 (13.0%)	0	−0.26 (−2.0 to 1.48)	0.7681	0.9601
	Hypercholesterolemia, *n* (%)	6 (28.6%)	19 (41.3%)	0	−0.47 (−1.63 to 0.69)	0.4269	0.7549
	Cardiovascular disease, *n* (%)	1 (4.8%)	9 (19.6%)	0	−1.71 (−3.92 to 0.5)	0.1297	0.6485
	Arterial hypertension, *n* (%)	9 (42.9%)	14 (30.4%)	0	0.66 (−0.49 to 1.81)	0.2586	0.7542
	Traumatic Brain Injury, *n* (%)	5 (23.8%)	6 (13.0%)	0	0.82 (−0.55 to 2.2)	0.2385	0.7542

We used regression models (linear and logistic). Data are given as mean and standard deviation (SD) for continuous clinical outcomes and as percentages for binary clinical outcomes. Models adjusted for sex, age at assessment, and disease duration. Beta (*β*) regression coefficients are given with the 95% CI. Statistically significant results are highlighted in bold with a * sign (*p* value < 0.05).

*p value* unadjusted *p* value, *adj p-value* corrected for multiple comparisons using FDR adjustment, *AAO* age at onset, *H&Y* Hoehn & Yahr, *MDS-UPDRS* Movement Disorders Society - Unified Parkinson’s Disease Rating Scale, *FOG* freezing of gait, *BDI* Beck Depression Inventory, *PDSS* Parkinson’s Disease Sleep Scale, *SCOPA-AUT* Scales for Outcomes in Parkinson’s Disease-Autonomic questionnaire, *SAS* Starkstein apathy scale, MoCA Montreal Cognitive Assessment, *ICD* impulse control disorder, *RBDSQ* REM Sleep Behavior Disorder Screening Questionnaire, *LEDD* L-dopa equivalent daily dose (mg/day), *PDQ-39* Parkinson’s Disease quality of life Questionnaire, *DBS* Presence of treatment by Deep Brain Stimulation.

### VUS and the glucosylceramidase structure

We detected nine already reported VUS (p.K13R, p.Y61H, p.R78C, p.L213P, p.E427K, p.A495P, p.H529R, p.R534C, p.T408T) and three new VUS (p.A97G, p.A215 and p.R434C).

**Fig. 4:**
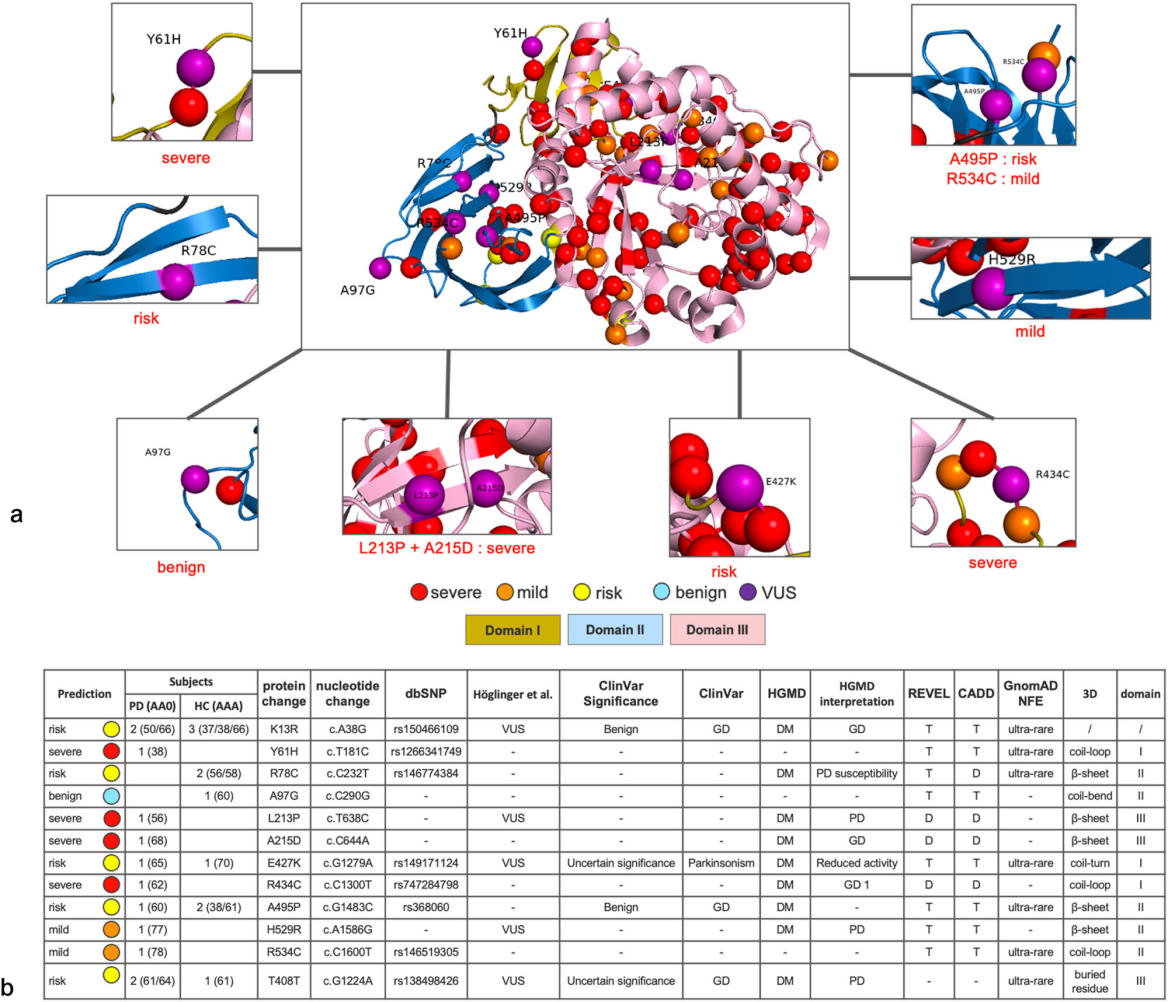
Sub-classification of VUS found in the Luxembourg Parkinson’s study. **a** *GBA1* missense and stop gain variants mapped onto the three-dimensional structure of GCase. Domain I is shown in dark yellow, domain II in blue, and domain III in pink. Domain I begins at residue 40 after the signal peptide sequence. Variants classified as severe are colored red, mild are colored orange, risk in yellow and VUS are colored purple. The 3D structure of GCase (PDB code 1ogs) was generated using PYMOL (http://www.pymol.org). **b** Proposed sub-classification of identified VUSs with their score in a known database. *GBA1* glucocerebrosidase gene, GD Gaucher’s disease, PD Parkinson’s disease, AAO age at onset, AAA age at assessment in visit1. HGMD The Human Gene Mutation Database, REVEL Rare Exome Variant Ensemble Learner, CADD Combined Annotation Dependent Depletion, gnomAD The Genome Aggregation Database. DM Disease causing mutation, D Deleterious, T Tolerate. Variants classified as severe are colored red, mild are colored orange, risk in yellow and VUS are colored purple.

According to our strategy developed for the VUS classification of *GBA1* variants, where we assign the pathogenicity based on the REVEL, the CADD and the dbscSNV scores, as well as the presence or absence of the variants in the patients. We propose to subclassify the VUS p.Y61H, p.L213P, p.A215D, and p.R434C as probably pathogenic severe variants (Supplementary Table 7). The variant p.L213P changes the leucine into proline, which is known to be the “helix breaker” amino acid that induces a bend into the protein structure^13^ (Supplementary Fig. 1). The p.L213P and p.A215D variants are in the catalytic site of the enzyme in the triose-phosphate isomerase (TIM) barrel structure. The p.Y61H variant (Fig. 4a) is located next to the residue position of the known severe variant p.C62W, and the patient carrying this variant had an AAO of 38 years, indicating an early-onset, probably severe form of PD. This patient has a family history of PD and reported that the paternal uncle and aunt were diagnosed with PD at the ages of 60 and 70, respectively. The p.R434C variant is close to a known severe (p.V433L) and mild (p.W432R, p.N435T) PD variants in the 3D structure. We compared the clinical scores obtained from carriers of known severe variants with the four carriers of probable severe VUS (p.Y61H, p.L213P, p.A215D, and p.R434C) (Supplementary Table 7). The z-score was used to determine the number of SD deviations from the mean for each clinical score. We observed that the PD patient carrying the p.L213P variant had a z-score that was significantly different for MDS-UPDRS II (z-score = 3.05) and MDS-UPDRS III (z-score = 2.94) confirming its classification as a severe variant.

We propose to subclassify the variants p.H529R and p.R534C as probably mild variants, as they are both found only in PD patients. The variants p.K13R, p.R78C, p.E427K, and p.A495P are subclassified as probable risk variants. The variant p.K13R is located in the signal peptide region. The variant p.R78C was annotated as “PD susceptibility” in HGMD with deleterious impact in CADD. The variant p.E427K was annotated as associated to “parkinsonism” in ClinVar and “reduced activity” in HGMD. We propose to classify the variant p.A97G as probably benign because it is localized in a coil-bend structure and is not close to any known pathogenic variants.

The synonymous variant p.T408T was found in two cases and one healthy control individual. Two established splice-site prediction scores (dbscSNV: ada_score 0.9797 and rf_score 0.85) agreed in their prediction that the variant is likely to affect splicing. HGMD classified the variant as disease mutation (DM) (Supplementary Table 4). Therefore, we propose to classify the variant as a risk variant.

Overall, we propose to classify four VUS variants as probably severe pathogenic variants (p.Y61H, p.L213P, p.A215D, and p.R434C), two as probably mild pathogenic variants (p.H529R and p.R534C), five as probably pathogenic risk variants (p.K13R, p.R78C, p.E427K, p.A495P, and p.T408T) and one as probably benign variants (p.A97G) (Fig. 4b).

## Discussion

Our study demonstrated in a large cohort the utility of targeted PacBio DNA sequencing for *GBA1* as a highly sensitive and specific method to identify known and novel *GBA1* variants and to overcome the problems posed by the presence of the *GBAP1* pseudogene by avoiding its amplification. The effectiveness of the targeted PacBio DNA sequencing method in investigating relevant genes with homologous pseudogenes has also been demonstrated in several other studies^13–16^. The PacBio method together with the WGS method combined with the new Gauchian tool showed a very high accuracy of 100% true positive validated variants. The comparative study that we performed with the different screening technologies to detect *GBA1* variants will help researchers to get a more accurate and comprehensive overview of *GBA1* variants. This implies a more critical evaluation of the results obtained by NeuroChip, which revealed a high proportion of false positive and negative results and those obtained by WGS, which will depend on the detection tool used for the complex *GBA1* region. Our study still has the limitation that we cannot fully exclude missing variants (false negatives) that could not be detected by all three methods used in our study. Long-read DNA sequencing excels in the detection of structural variants. However, the method employed in this study relies on a single amplicon, limiting its efficiency in detecting structural variants due to the generation and purification of amplicons of specific sizes only. However, we would like to highlight the fact that the PacBio-based method can be a cost-effective (≃30€_/sample_ for PacBio) alternative for the high-fidelity calling of *GBA1* variants. *GBA1* variants have been identified as the most common genetic risk factor for the development of PD. *GBA1* variants have typically been observed in 4%–12% of PD patients in different populations worldwide, with the highest prevalence of 20% described in Ashkenazi Jewish PD patients^17,18^. Large differences of prevalence were observed depending on the ethnicity of the cohort, the variants studied, and the sequencing method used. Previous studies looking only at coding regions reported frequencies of 14.3% in Italians^19^ (*n* = 874), 11.7% in southern Spanish^20^ (*n* = 532), 9.2% in New Zealanders of European descent^5^ (*n* = 229) and 8.3% in Irish^21^ (*n* = 314) (Supplementary Table 8). Our study describes the landscape of *GBA1* carriers in the Luxembourgish population showing a high prevalence (12.1%) of *GBA1* variants that could be the major genetic risk factor of PD in Luxembourg. Moreover, we observed a significantly higher proportion of pathogenic (severe, mild and risk) *GBA1* variants in PD patients compared to HC (10.4% vs 4.3%; OR = 2.6; CI = [1.6,4.1], *p* = 0.0001). Compared to previous studies, our study highlights that using the new PacBio sequencing method, the Luxembourg Parkinson’s study showed a comparable frequency of PD_*GBA1*_ carriers reported so far in similarly sized Italian^19^ and Spanish^20^ cohorts (Supplementary Table 8). When comparing previous reports of *GBA1* variants in different populations, we want to highlight the fact that only cohorts that used full Sanger sequencing were able to detect the RecNciI recombinant allele so far. This once more emphasizes the accuracy of the PacBio sequencing methods for detecting rare and complex *GBA1* variants. Additionally, we confirmed that severe variants showed a higher OR than risk variants, which supports the concept of graded risk for different *GBA1* variants in PD_*GBA1*_ carriers^20^.

The most prevalent *GBA1* variant in the Luxembourg Parkinson’s study was p.E365K, and the frequency of this variant was similar to what was described in the Irish^21^, Spanish^20^, and New Zealand^5^ populations. It is interesting to note that homozygous carriers of the p.E365K variant do not develop GD^22^. This variant is associated with PD, and multiple studies have found enrichments varying from 1.60 to 3.34^23–25^. Furthermore, carriers of the risk variants p.E365K and p.T408M could be associated with atypical parkinsonism, as these variants were the only ones also present in patients with DLB and PSP in our cohort. Whether this is simply related to the higher frequency of these risk variants in the general population or does have a specific impact on the phenotype needs to be determined in larger studies focusing on *GBA1* variants in atypical parkinsonism^26^.

We present a concept for classifying VUS in the *GBA1* gene according to the localization in relation to known variants in sequence and 3D structure, which may help to provide access to future targeted therapies for these patients. Here additional in vitro and ex vivo studies are needed to functionally validate the impact of these VUS on GCase function in neurons derived from stem cells or in enzyme-activity assays in cerebrospinal fluid of affected carriers of these VUS.

Additionally, we observed that the average AAO in PD was about four years younger in severe *GBA1* carriers compared to non-*GBA1* carriers. This was also observed in previous studies, which showed that PD_*GBA1*_ patients generally have an earlier AAO compared to non-carriers with a median onset in the early fifties^27,28^.

Recent studies have shown that PD_*GBA1*_ carriers have a higher prevalence of cognitive impairment^19,29,30^ and non-motor symptoms including neuropsychiatric disturbances^19,20^, autonomic dysfunction^29^, and sleep disturbances such as RBD^31^. Although not significant after *p* value adjustment, we found a similar trend and noticed that motor symptoms such as gait disorder, non-motor symptoms such as depression and hallucinations, were associated with a more aggressive clinical phenotype in severe *GBA1* carriers, supporting the effect of differential *GBA1* variant severity^20,32^.

In conclusion, this study showed the utility of targeted PacBio DNA sequencing to identify known and novel *GBA1* variants with high accuracy. These findings offer important access to variant-specific counseling. Furthermore, our study describes the full landscape of *GBA1*-related PD in the current Luxembourgish population showing the high prevalence of *GBA1* variants as the major genetic risk in PD.

## Methods

### Clinical cohort

At the time of analysis, the Luxembourg Parkinson’s study comprised 1568 participants (760 patients of parkinsonism and 808 healthy controls (HC) in the frame of the National Centre for Excellence in Research on Parkinson’s disease program (NCER-PD).

All patients complied with the diagnostic criteria of typical PD or atypical parkinsonism as assessed by neurological examination following the United Kingdom Parkinson’s Disease Society Brain Bank (UKPDSBB) diagnostic criteria^33^: 660 fulfilled the criteria for PD, 60 for progressive supranuclear palsy (PSP) including corticobasal syndrome as a subtype of PSP (PSP-CBS), 25 for Dementia with Lewy Body (DLB), 14 for Multiple System Atrophy, and one for Fronto-temporal dementia with parkinsonism. All patients and HC underwent a comprehensive clinical assessment of motor and non-motor symptoms, neuropsychological profile and medical history along with comorbidities. The clinical symptoms assessed, and scales applied are defined in the Supplemental Information^34^. All individuals provided written informed consent. The patients were reassessed at regular follow-up visits every year and the HC every 4 years. We considered early-onset PD patients those with AAO equal to or younger than 45 years^35^. The genotype-phenotype analysis was based on the assessment of the first visit. The final diagnosis was taken according to the last visit. The study has been approved by the National Research Ethics Committee (CNER Ref: 201407/13 and 202304/03).

### NeuroChip array

Genotyping was carried out on the InfiniumR NeuroChip Consortium Array^9^ (v.1.0 and v1.1; Illumina, San Diego, CA USA). For rare variants analysis, standard quality control (QC) procedures were conducted, using PLINK v1.9^36^, to remove variants if they had a low genotyping rate (<95%) and Hardy-Weinberg equilibrium *p* value < 1 × 10^−6^. As an additional quality filter, we applied a study-wide allele frequency threshold of <1% in our cohort for rare variants. For further statistical analysis, we excluded individuals of non-European ancestry using PLINK 1.9 multidimensional scaling and merged our data with the 1000 genomes dataset^37^. We selected only samples of European ancestry excluding those with > ±3 SD based on the first and the second principal components.

### *GBA1*-targeted PacBio DNA long-read amplicon sequencing

The targeted *GBA1* gene screening was performed by single-molecule real-time (SMRT) long read sequencing^8^ using Sequel II instrument (PacBio). The targeted *GBA1* gene coordinates were chr1:155,232,501-155,241,415 (USCS GRCh38/hg38). Long-distance PCR was performed using *GBA1*-specific primer sequences (Forward: 5′-GCTCCTAAAGTTGTCACCCATACATG-3′ and Reverse: 5′-CCAACCTTTCTTCCTTCTTCTCAA-3′)^38^ and the 2x KAPA HiFi Hot Start ReadyMix (Roche), which avoid *GBAP1* pseudogene amplification. For sample multiplexing, dual asymmetric barcoding was used based on a different 16-bp long index sequence upstream of each of the reverse and forward primers to allow the generation of uniquely barcoded amplicons in one-step PCR amplification. QC was performed prior to pooling. Pools of amplicons were purified with AMPure PacBio beads. A total of 1700 ng of purified amplicon pool was used as input for the SMRTbell library using the SMRTbell Express Template Prep Kit 2.0 (PacBio). Binding of the polymerase and diffusion loading on SMRTCell 8 M was prepared according to SMRTLink instructions with CCS reads as sequencing mode (version SMRT Link: 9.0.0.92188). We generated high-quality consensus reads using the PacBio Sequel II sequencer on Circular Consensus Sequencing mode using the pbccs (v6.0.0) tool. The method replicates both strands of the target DNA^39^. We demultiplexed and mapped reads from each sample to the human reference genome GRCh38 using minimap2^40^ from the pbmm2 package (v1.4.0) (https://github.com/PacificBiosciences/pbmm2). We used the MultiQC^41^ tool and selected samples with more than 30-fold coverage. For variant calling, we used the DeepVariant^42^ (1.0) with models optimized for CCS reads. Finally, we selected variants with quality above 30 (QUAL > 30).

### Whole genome sequencing

The TruSeq Nano DNA Library Prep Kit (Illumina, San Diego, CA, USA) and MGIEasy FS DNA Prep kit (BGI, China) were used according to the manufacturer’s instructions to construct the WGS library. Paired-end sequencing was performed with the Illumina NovaSeq 6000^43^ and on the MGI G400 sequencers. A QC of the raw data was performed using FastQC (version 0.11.9: http://www.bioinformatics.babraham.ac.uk/projects/fastqc/). To call the variants, we used the Bio-IT Illumina Dynamic Read Analysis for GENomics (DRAGEN) DNA pipeline^44^ v4^45^ with standard parameters and with or without the ‘*GBA* caller’ option, which uses the Gauchian tool. To select the high-quality variants, we annotated and selected variants using VariantAnnotator and SelectVariants modules of the Genome Analysis Toolkit (GATK 4)^46^ pipeline and applied the following additional filtering steps: VariantFiltration module for SNVs (QD < 2, FS > 60, MQ < 40, MQRankSum < −12, ReadPosRankSum < −8, DP < 10.0, QUAL < 30, VQSLOD < 0, ABHet > 0.75 or <0.25, SOR > 3 and LOD < 0), and insertions-deletions (QD < 2, FS > 200, QUAL < 30, ReadPosRankSum < −20, DP < 10 and GQ_MEAN < 20).

### Variant annotation and validation

Variant annotation was done with ANNOVAR^47^, using the Genome Aggregation Database (gnomAD r2.1)^48^, the Human Gene Mutation Database (HGMD)^49^ and ClinVar^50^, and the Combined Annotation Dependent Depletion (CADD)^51^ and Rare Exome Variant Ensemble Learner (REVEL)^52^ to score the pathogenicity of missense variants^53^. For variants in splice sites, we used the ada_score and rf_score from dbscSNV (version 1.1)^54^. Ada_score ≥ 0.6 or rf_score ≥ 0.6 indicate that the variant is likely to affect splicing.

Rare variants were selected according to MAF < 1% in gnomAD for the Non-Finnish European (NFE) population in the ‘non-neuro’ gnomAD subset. Then, exonic and splicing variants (±2 bp from the exon boundary) were selected for autosomal dominant (*LRRK2*, *SNCA*, *VPS35*, *GBA1*) and autosomal recessive (*PRKN*, *PINK1*, *PARK7*, *ATP13A2*) PD genes. Rare variants within these genes were then confirmed by Sanger sequencing^55^.

### CNVs in PD genes

To detect the presence of copy number variants (CNVs) in selected six PD genes (*PARK7*, *ATP13A2*, *PINK1*, *SNCA*, *GBA1*, and *PRKN*), we used the PennCNV tool (v1.0.5)^56^ using the Neurochip array data applying the same filtering steps as previously described for CNV calls in PD^11^. The multiplex-ligation dependent probe amplification method, which exclusively targets the selected genes, was used to validate the CNVs. Six patients with each one CNVs in one of the six PD genes were found and no CNV in *GBA1* was found. To detect CNVs within the *GBA1* gene through the analysis of PacBio data, we employed the pbsv tool (version 2.9.0) (https://github.com/PacificBiosciences/pbbioconda), which is specifically designed for long-read data analysis from PacBio. This tool successfully identifies 59.46% of structural variants with precision^57,58^.

### *GBA1* variant nomenclature

All variants in *GBA1* were annotated based on GRCh37 and were numbered according to the current variant nomenclature guidelines (http://varnomen.hgvs.org), based on the primary translation product (NM_001005742), which includes the 39-residue signal peptide.

### *GBA1* variant classification

*GBA1* variants classification was done according to the PD literature based on the work of Höglinger and colleagues in 2022^12^. Exonic or splice-site variants that are not mentioned in the paper were subclassified as “severe” *GBA1* variants if they were annotated as pathogenic in ClinVar, otherwise they were subclassified as variants with unknown significance (VUS)^51^.

### Statistical analysis

To assess the frequency of different *GBA1* variant types and to analyze the genotype–phenotype associations in the Luxembourg Parkinson’s Study, we considered only unrelated individuals and kept only one proband per family. For cases, we kept the patient with the earliest AAO. To account for age-dependent penetrance, we excluded HC with first-degree relatives (parents, sibs, and offspring) with PD and an AAA of less than 60 years. This reduced the age difference between cases and HC. We also excluded carriers of rare variants or CNVs in PD-associated genes (except *GBA1*) and individuals of non-European ancestry. Thus, 1410 unrelated individuals (735 patients and 675 HC) were selected for the statistical analysis.

We used regression models to assess the effect of PD_*GBA1*_ carrier status on the clinical variables. In these models, the dependent variable was the clinical outcome, while the predictor was *GBA1* carrier status. We excluded individuals carrying only VUS or synonymous variants. To this aim, we performed three types of association tests: (1) all PD_*GBA1*_ pathogenic variant carriers (severe, mild and risk) vs. PD_*GBA1*-non-carriers_, (2) for each sub-group of PD_*GBA1*_ pathogenic variant carriers vs. PD_*GBA1*-non-carriers_, (3) severe PD_*GBA1*_ pathogenic variant carriers *vs* combined mild and risk PD_*GBA1*_ pathogenic variant carriers. The effect of each factor was expressed as the Beta (*β*) regression coefficient. The odds ratio (OR) along with a 95% confidence interval (CI) was used to assess whether a given exposure was a risk factor for a given outcome. Regression models were adjusted for AAA, sex, and disease duration. FDR-adjusted *p* value < 0.05 was considered as statistically significant.

### Structure-based evaluation of VUS

To evaluate VUS variants, we implemented a method to assign the pathogenicity based on the REVEL^53^ and CADD^51^ scores for missense variants and the dbscSNV scores (ada_score and rf_score) for splice variants according to the dbNFSP^54^ definition, as well as whether the patients carried the variants. We reclassified a VUS (1) as “severe” if the variant was present only in patients and with deleterious effect in all scores or present only in patients with early-onset PD, (2) as “mild” if the variant was present only in patients and with tolerated effect in all scores, (3) as “risk” if present in patients and HCs or with tolerated and deleterious effect in either score or annotated as “PD susceptibility” in HGMD, and (4) as “benign” if present only in HC.

We mapped the known pathogenic missense variants and newly identified VUS in our cohort together with all reported population variants from gnomAD onto the *GBA1* protein sequence and the 3D structure. We used the X-ray structure of GCase at 2.0 Å resolution (PDB structure accession code 1ogs; https://www.rcsb.org/) (Supplementary Fig. 2). Analysis of the 3D structure was carried out using PyMOL (http://www.pymol.org). VUS were evaluated as a risk variant if they were 2 bp positions away in sequence or had a C-alpha distance of less than 5 Å in 3D from another known pathogenic variant similar to the approach used by Johannesen et al.^59^.

### Supplementary information

Supplemental material

## Data Availability

The dataset for this manuscript is not publicly available as it is linked to the Luxembourg Parkinson’s Study and its internal regulations. Any requests for accessing the dataset can be directed to request.ncer-pd@uni.lu.

## Code availability

The code behind the analyses is available under the Apache-2.0 license. More information on how to request access to the data and the code is available at 10.17881/eqzd-a607.
