# Author Correction: Accurate long-read sequencing identified *GBA1* as major risk factor in the Luxembourgish Parkinson’s study

**DOI:** 10.1038/s41531-023-00613-x

**Published:** 2023-12-18

**Authors:** Sinthuja Pachchek, Zied Landoulsi, Lukas Pavelka, Claudia Schulte, Elena Buena-Atienza, Caspar Gross, Ann-Kathrin Hauser, Dheeraj Reddy Bobbili, Nicolas Casadei, Patrick May, Rejko Krüger, on behalf of the NCER-PD Consortium, Geeta Acharya, Gloria Aguayo, Myriam Alexandre, Muhammad Ali, Wim Ammerlann, Giuseppe Arena, Rudi Balling, Michele Bassis, Roxane Batutu, Katy Beaumont, Regina Becker, Camille Bellora, Guy Berchem, Daniela Berg, Alexandre Bisdorff, Ibrahim Boussaad, David Bouvier, Kathrin Brockmann, Jessica Calmes, Lorieza Castillo, Gessica Contesotto, Nancy De Bremaeker, Nico Diederich, Rene Dondelinger, Nancy E. Ramia, Daniela Esteves, Guy Fagherazzi, Jean-Yves Ferrand, Katrin Frauenknecht, Manon Gantenbein, Thomas Gasser, Piotr Gawron, Soumyabrata Ghosh, Marijus Giraitis, Enrico Glaab, Martine Goergen, Elisa Gómez De Lope, Jérôme Graas, Mariella Graziano, Valentin Groues, Anne Grünewald, Wei Gu, Gaël Hammot, Anne-Marie Hanff, Linda Hansen, Michael Heneka, Estelle Henry, Sylvia Herbrink, Sascha Herzinger, Michael Heymann, Michele Hu, Alexander Hundt, Nadine Jacoby, Jacek Jaroslaw Lebioda, Yohan Jarosz, Sonja Jónsdóttir, Quentin Klopfenstein, Jochen Klucken, Rejko Krüger, Pauline Lambert, Roseline Lentz, Inga Liepelt, Robert Liszka, Laura Longhino, Victoria Lorentz, Paula Cristina Lupu, Tainá M. Marques, Clare Mackay, Walter Maetzler, Katrin Marcus, Guilherme Marques, Patricia Martins Conde, Deborah Mcintyre, Chouaib Mediouni, Francoise Meisch, Myriam Menster, Maura Minelli, Michel Mittelbronn, Brit Mollenhauer, Friedrich Mühlschlegel, Romain Nati, Ulf Nehrbass, Sarah Nickels, Beatrice Nicolai, Jean-Paul Nicolay, Fozia Noor, Marek Ostaszewski, Clarissa P. C. Gomes, Claire Pauly, Laure Pauly, Lukas Pavelka, Magali Perquin, Rosalina Ramos Lima, Armin Rauschenberger, Rajesh Rawal, Kirsten Roomp, Eduardo Rosales, Isabel Rosety, Estelle Sandt, Stefano Sapienza, Venkata Satagopam, Margaux Schmitt, Sabine Schmitz, Reinhard Schneider, Jens Schwamborn, Raquel Severino, Amir Sharify, Ekaterina Soboleva, Kate Sokolowska, Hermann Thien, Elodie Thiry, Rebecca Ting Jiin Loo, Christophe Trefois, Johanna Trouet, Olena Tsurkalenko, Michel Vaillant, Mesele Valenti, Gilles Van Cutsem, Carlos Vega, Liliana Vilas Boas, Maharshi Vyas, Richard Wade-Martins, Paul Wilmes, Evi Wollscheid-Lengeling, Gelani Zelimkhanov

**Affiliations:** 1 LCSB, Luxembourg Centre for Systems Biomedicine, University of Luxembourg, Esch-Sur-Alzette, Luxembourg; 2 Parkinson Research Clinic, Centre Hospitalier de Luxembourg (CHL), Luxembourg, Luxembourg; 3 Transversal Translational Medicine, Luxembourg Institute of Health (LIH), Strassen, Luxembourg; 4 Department of Neurodegeneration, Center of Neurology, Hertie Institute for Clinical Brain Research, German Center for Neurodegenerative Diseases, University of Tübingen, Tübingen, Germany; 5 Institute of Medical Genetics and Applied Genomics, University of Tübingen, Tübingen, Germany; 6 NGS Competence Center Tübingen (NCCT), University of Tübingen, Tübingen, Germany; 7 Luxembourg Institute of Health, Strassen, Luxembourg; 8 Centre Hospitalier de Luxembourg, Strassen, Luxembourg; 9 Center of Neurology and Hertie Institute for Clinical Brain Research, Department of Neurodegenerative Diseases, University Hospital Tübingen, Tübingen, Germany; 10 Centre Hospitalier Emile Mayrisch, Esch-sur-Alzette, Luxembourg; 11 Laboratoire National de Santé, Dudelange, Luxembourg; 12 Association of Physiotherapists in Parkinson’s Disease Europe, Esch-sur-Alzette, Luxembourg; 13 Faculty of Science, Technology and Medicine, University of Luxembourg, Esch-sur-Alzette, Luxembourg; 14 Department of Epidemiology, CAPHRI School for Public Health and Primary Care, Maastricht University Medical Centre+, Maastricht, The Netherlands; 15 Centre Hospitalier du Nord, Ettelbrück, Luxembourg; 16 Oxford Parkinson’s Disease Centre, Nuffield Department of Clinical Neurosciences, University of Oxford, Oxford, UK; 17 Private practice, Ettelbruck, Luxembourg; 18 Parkinson Luxembourg Association, Leudelange, Luxembourg; 19 Westpfalz-Klinikum GmbH, Kaiserslautern, Germany; 20 Oxford Centre for Human Brain Activity, Wellcome Centre for Integrative Neuroimaging, Department of Psychiatry, University of Oxford, Oxford, UK; 21 Department of Neurology, University Medical Center Schleswig-Holstein, Kiel, Germany; 22 Ruhr-University of Bochum, Bochum, Germany; 23 Paracelsus-Elena-Klinik, Kassel, Germany; 24 Private practice, Luxembourg, Luxembourg; 25 Oxford Parkinson’s Disease Centre, Department of Physiology, Anatomy and Genetics, University of Oxford, South Parks Road, Oxford, UK

**Keywords:** Parkinson's disease, Parkinson's disease

Correction to: *npj Parkinson’s Disease* 10.1038/s41531-023-00595-w, published online 23 November 2023

In this article the wrong Table appeared as Table 3.; the Table should have appeared as shown below. The original article has been corrected.

The following text that describes data from the previous version of Table 3 has also been removed: “Compared to severe (6.4 ± 4.7) and risk (4.4 ± 4.9) variant carriers, the mild (1.7 ± 1.4) variant carriers show a significantly shorter disease duration (Table 3).”

**Table:** 

Features	All pathogenic variants (*n* = 67)	Severe (*n* = 21)	Mild (*n* = 7)	Risk (*n* = 39)	Non carriers (*n* = 554)
AAA, mean (SD)	66.5 (±10.2) [OR = 0.31; *p* = 0.3977]	65.1 (±10.2) [OR = 0.08; *p* = 0.292]	67.1 (±15.6) [OR = 0.59; *p* = 0.8959]	67.1 (±9.2) [OR = 0.57; *p* = 0.7512]	67.6 (±10.7)
Sex, Male n (%)	40 (59.7%) [OR = 0.71; *p* = 0.1912]	13 (61.9%) [OR = 0.78; *p* = 0.5795]	5 (71.4%) [OR = 1.19; *p* = 0.8336]	22 (56.4%) [OR = 0.62; *p* = 0.151]	375 (67.7%)
AAO, mean (SD)	61.6 (±11.5) [OR = 0.35; *p* = 0.484]	58.6 (±13.1) [OR = 0.02; *p* = 0.1158]	65.4 (±17.0) [OR = 16.16; *p* = 0.5308]	62.5 (±9.3) [OR = 0.9; *p* = 0.9548]	62.6 (±11.6)
AAO < 45, N (%)	8 (11.9%) [OR = 1.74; *p* = 0.1767]	**5 (23.8%)** **[OR** **=** **4.02;** ***p*** **=** 0.0098*]	2 (28.6%) [OR = 5.14; *p* = 0.0549]	1 (2.6%) [OR = 0.34; *p* = 0.2907]	40 (7.2%)
Disease Duration, mean (SD)	4.7 (±4.8) [OR = 0.79; *p* = 0.7303]	6.4 (±4.7) [OR = 4.07; *p* = 0.2238]	1.7 (±1.4) [OR = 0.04; *p* =0.0981]	4.4 (±4.9) [OR = 0.57; *p* = 0.5103]	5.0 (±5.2)
Family History, N (%)	**25 (37.3%)** **[OR** **=** **1.74;** ***p*** **=** 0.0401*]	8 (38.1%) [OR = 1.8; *p* = 0.2001]	2 (28.6% [OR = 1.17; *p* = 0.8508]	15 (38.5%) [OR = 1.83; *p* = 0.0782]	141 (25.5%)

