# Supplementary material for: Accurate long-read sequencing identified GBA1 as major risk factor in the Luxembourgish Parkinson’s study
Source: NPJ Parkinsons Dis. 2023 Nov 23;9:156. doi: 10.1038/s41531-023-00595-w (PMC10667262; doi:10.1038/s41531-023-00595-w)
Supplement: Supplementary file 1 — Supplemental material [file 41531_2023_595_MOESM1_ESM.pdf]

Supplementary material

Supplementary notes. Clinical symptoms and scales

Clinical symptoms and scales Movement Disorder Society-Unified Parkinson's disease Rating Scale (MDS-UPDRS I-IV) and Scales for Outcomes in Parkinson's Disease-Autonomic questionnaire (SCOPA-AUT) are used under the license number (14017_ND). Probable REM sleep behaviour disorder (pRBD) was based on the validated self-reporting questionnaire REM Sleep Behaviour Disorder Screening Questionnaire (RBDSQ) where possible pRBD was defined as RBDSQ ≥ 7[1]. Assessment of sleep quality was done via Parkinson's Disease Sleep Scale (PDSS)[2]. The calculation of levodopa equivalent daily dose (LEDD, reported in g/day) was based on established conversion factors[3]. Definition of constipation corresponds to the diagnostic criteria ROME III and information was acquired in a semi-structured interview[4]. The Hoehn and Yahr scale (H&Y) corresponds to the modified version of the scale[5]. Quality of life was assessed via Parkinson's disease Questionnaire 39 (PDQ-39)[6]. Depression symptoms were reflected by Beck Depression Inventory Version I (BDI)[7]. Olfactory function was examined with 16 items Sniffin' Stick test[8]. Cognitive performance was assessed via Montreal Cognitive Assessment (MoCA)[9]. Presence of recurrent orthostatic hypotension was assessed using a semi-structured interview inquiring for the symptoms of orthostatic hypotension, i.e. faintness, dizziness, light-headedness, vertigo, hearing disturbance, visual disturbance or syncope following the tilting, standing up or after a long standing relieved by sitting down or laying down. Symptoms included in the analysis (gait disorder, falls, freezing of gait (FOG), dyskinesia, motor fluctuations, excessive daily sleepiness, insomnia, dysphagia, urinary incontinence (corresponding to any type of urinary incontinence, i.e. stress, urge, overflow or mixed urinary incontinence), hallucinations and impulse control disorder (ICD)) were assessed during a semi-structured interview of the participant and/or the participant's proxy with a study physician and refer to the current motor and non-motor symptoms at the time of assessment. DBS, Presence of treatment by Deep Brain Stimulation. Restless leg syndrome corresponds to history or presence of rest less leg syndrome based on the accurate anamnestic description corresponding to the diagnostic features/criteria.

Supplementary Table 1. Comparison between AAO, AAA and Gender for the Luxembourgish cohort before and after the exclusion criteria.

	Before		After	
**Features**	**Cases (n = 760)**	**Controls (n = 808)**	**Cases (n = 735)**	**Controls (n = 675)**
**AAA**	67.6 (±10.5)	59.3 (±12.2)	67.9 (±10.4)	61 (±11.5)
**AAO, mean (SD)**	63 (±11.5)	-	63.2 (±11.3)	-
**Sex, Male % (n)**	505 (66.4%)	426 (52.7%)	489 (66.5%)	368 (54.5%)

We applied a t-test for AAA and AAO features and a Fisher test for the gender features. Exclusion criteria : the first-degree family members interrelated in the cohort, the healthy controls of young age of assessment (< 60 AAA) with first-degree PD relatives, the CNV carriers*,* carrier of PD-causing variants and the ethnic outliers.

Supplementary Table 2. PCR primers

PCR Primers targeted *GBA1* gene (Stone et al. 2000)

exon	Forward	reverse	length
1–5	CCTAAAGTTGTCACCCATAC	AGCAGACCTACCCTACAGTTT	2972
5–7	GACCTCAAATGATATACCTG	AGTTTGGGAGCCAGTCATTT	2049
8–11	TGTGTGCAAGGTCCAGGATCAG	ACCACCTAGAGGGGAAAGTG	1682

Nested Sequencing Primers targeted *GBA1* gene (partly Stone et al.2000)

exon	Forward	Reverse	Length
1	CCTAAAGTTGTCACCCATAC	aaattccagtgccaggattc	392
2	GAGAGTAGTTGAGGGGTGGA	CAAAGGACTATGAGGCAGAA	210
3	ATGTGTCCATTCTCCATGTC	GGTGATCACTGACACCATTT	323
4	GGTGTCAGTGATCACCATGG	ACGAAAAGTTTCAATGGCTCT	263
5	GCAAGTGATAAGCAGAGTCC	AGCAGACCTACCCTACAGTTT	280
6	CTCTGGGTGCTTCTCTCTTC	ACAGATCAGCATGGCTAAAT	271
7	TTGGCCGGATCATTCATGAC	AGTTTGGGAGCCAGTCATTT	342
8	TGTGTGCAAGGTCCAGGATCAG	TTTGCAGGAAGGGAGACTGG	294
9	CACAGGGCTGACCTACCCAC	GCTCCCTCGTGGTGTAGAGT	307
10	CAGGAGTTATGGGGTGGGTC	GAGGCACATCCTTAGAGGAG	329
11	GTGGGCTGAAGACAGCGTTGG	ACCACCTAGAGGGGAAAGTG	342

The first step is a long-range PCR which ensures that the pseudogene of *GBA1* is not amplified.

Supplementary Table 3. Exonic and splice-site *GBA1* variants found in the Luxembourg Parkinson's study.

Subclassification	protein change	Nucleotide change	dbSNP	Variant type	Höglinger et al.	ClinVar Significance	ClinVar	HGMD	REVEL	CADD	gnomAD NFE	3D	domain
severe	-	c.115+1G>A	rs104886460	splicing	severe	Pathogenic	PD/GD/DLB	DM	-	D	rare	/	/
	p.P161S	c.C481T	rs121908299	missense	-	Pathogenic	GD	DM	D	D	-	β-sheet	III
	p.G234W	c.G700T	-	missense	severe	-	-	DM	D	D	-	coil-loop	III
	p.G241R	c.G721A	rs409652	missense	severe	Pathogenic	GD	DM	D	T	rare	coil-turn	III
	p.F252I	c.T754A	rs381737	missense	severe	Pathogenic	GD	DM	D	T	rare	α-helix	III
	p.H294Q	c.T882G	rs367968666	missense	severe	Pathogenic	GD	DM	T	T		α-helix	III
	p.R398*	c.C1192T	rs121908309	nonsense	severe	Pathogenic	GD	DM	-	D	-	α-helix	III
	p.G416S	c.G1246A	rs121908311	missense	severe	Pathogenic	GD	DM	D	T	rare	β-sheet	III
	p.L483P*	c.T1448C	rs421016	missense	severe	Pathogenic	GD	DM	D	T	rare	β-sheet	II
	p.R502H	c.G1505A	rs80356772	missense	severe	Conflicting interpretations of pathogenicity	GD	DM	D	D	rare	coil-loop	II
mild	p.N409S	c.A1226G	rs76763715	missense	mild	Pathogenic	PD/GD/DLB	DM	T	T	rare	α-helix	III
risk	p.E365K	c.G1093A	rs2230288	missense	risk	Benign	GD	DM	T	T	1.4%	α-helix	III
	p.T408M	c.C1223T	rs75548401	missense	risk	Conflicting interpretations of pathogenicity	PD/GD	DM	T	T	1.1%	buried residue	III
VUS	p.K13R	c.A38G	rs150466109	missense	VUS	Benign	GD	DM	T	T	rare	/	/
	p.Y61H	c.T181C	rs1266341749	missense	-	-	-	-	T	T	rare	coil-loop	I
	p.R78C	c.C232T	rs146774384	missense	-	-	-	DM	T	D	rare	β-sheet	II
	p.A97G	c.C290G	-	missense	-	-	-	-	T	T	-	coil-bend	II
	p.L213P	c.T638C	-	missense	VUS	-	-	DM	D	D	-	β-sheet	III
	p.A215D	c.C644A	-	missense	-	-	-	DM	D	D	-	β-sheet	III
	p.E427K	c.G1279A	rs149171124	missense	VUS	Uncertain significance	Parkinsonism	DM	T	T	rare	coil-turn	I
	p.R434C	c.C1300T	rs747284798	missense	-	-	-	DM	D	D	-	coil-loop	I
	p.A495P*	c.G1483C	rs368060	missense	-	Benign	GD	DM	T	T	rare	β-sheet	II
	p.H529R	c.A1586G	-	missense	VUS	-	-	DM	T	T	-	β-sheet	II
	p.R534C	c.C1600T	rs146519305	missense	-	-	-	-	T	T	rare	coil-loop	II
	p.T408T	c.G1224A	rs138498426	synonymous	VUS	Uncertain significance	GD	DM	-	-	rare	buried residue	III

Abbreviations: *GBA1*, glucocerebrosidase gene; GD, Gaucher's disease; PD, Parkinson's disease; DLB, Dementia with Lewy Bodies. HGMD, The Human Gene Mutation Database; REVEL, Rare Exome Variant Ensemble Learner; CADD, Combined Dependent Depletion; gnomAD, The Genome Aggregation Database. DM, Disease causing mutation; D, Deleterious; T, Tolerate; VUS, Variants of unknown significan; **RecNcil* (p.L483P; p.A495P; p.V499V).

Supplementary Table 4. Exonic synonymous *GBA1* variants in the Luxembourg Parkinson's study.

Subjects		nucleotide change	protein change	dbSNP	Exon	3D	gnomAD NFE	ClinVar	ClinVar Significance	HGMD
PD	HC									
5	3	c.G1497C	p.V499V*	rs1135675	11	β-sheet	rare	-	-	-
	1	c.C1473A	p.P491P	rs149257166	11	coil-turn	-	-	-	-
1		c.A1455G	p.A485A	rs199928507	11	β-sheet	rare	-	-	-
1		c.C228T	p.F76F	rs75954905	4	β-sheet	rare	-	-	-
2	1	c.G1224A	p.T408T	rs138498426	9	buried residue	rare	GD	Uncertain significance	PD
1		c.T1029C	p.Y343Y	-	9	coil-turn	-	-	-	-
2		c.C630T	p.P210P	rs201615998	7	coil-loop	rare	-	-	-
1		c.C585G	p.L195L	rs1157873928	6	π-helix	-	-	-	-
1		c.G105A	p.S35S	rs148001886	3	-	rare	-	-	-

All variants were identified in the heterozygous state.
Abbreviations: *GBA1*, glucocerebrosidase gene; GD, Gaucher's disease; PD, Parkinson's disease; HC, Healthy controls; HGMD, The Human Gene Mutation Database ; gnomAD, The Genome Aggregation Database; **RecNcil* (p.L483P; p.A495P; p.V499V).

Supplementary Table 5. Splicing, intronic and UTRs regions variants detected in the Luxembourg Parkinson's study by PacBio sequencing method.

N°	POS	REF	ALT	Region	Transcript	Nucleotide changes	GnomAD NFE	avsnp150	ClinVar	ClinVar Significance	PD	HC
1	155204345	C	T	UTR3	NM_001005742	c.*441G>A					1	0
2	155204541	G	A	UTR3	NM_001005743	c.*245C>T	0.0002				0	1
3	155204621	A	G	UTR3	NM_001005744	c.*165T>C	0.0393	rs375776699			81	79
4	155204684	A	G	UTR3	NM_001005745	c.*102T>C	0.0007	rs368275143			17	21
5	155204694	C	T	UTR3	NM_001005746	c.*92G>A	0.0025	rs708606	GD/DLB	Likely_benign	18	23
6	155204701	C	T	UTR3	NM_001005747	c.*85G>A					0	1
7	155205200	G	T	intronic			0.0018	rs183510604			0	2
8	155205203	G	A	intronic			0.0003	rs426516			4	1
9	155205300	G	A	intronic							3	1
10	155205359	G	A	intronic							0	1
11	155205378	C	T	intronic			0.0139	rs12752133			30	16
12	155205646	G	C	intronic			6.668e-05				1	0
13	155205669	G	T	intronic			0.9992	rs3115534	GD	Benign	752	806
14	155205709	A	G	intronic			0.0007	rs548435731			0	1
15	155205748	G	A	intronic			6.681e-05	rs1003268223			1	1
16	155205801	A	G	intronic							1	0
17	155205964	C	T	intronic							0	1
18	155206430	G	A	intronic			6.688e-05	rs767239225			0	1
19	155206542	TTGTGTGTGTA	T	intronic				rs998227221			0	1
20	155206578	G	GTA	intronic			0.0003	rs200655080			1	3
21	155206580	A	G	intronic			6.711e-05	rs146697312			1	0
22	155206863	C	G	intronic			6.704e-05	rs1026559493			1	0
23	155206981	C	T	intronic							1	0
24	155207030	G	A	intronic			0.0233	rs72704130			36	52
25	155207050	G	A	intronic				rs749925127			1	0
26	155207387	A	T	intronic			0.015	rs140335079	PD/GD/DLB	Benign	14	27
27	155207449	C	T	intronic				rs1006437355			2	0
28	155207550	G	T	intronic			0.0001				0	2
29	155207674	C	CAG	intronic							1	0
30	155207733	C	T	intronic			0.0396	rs28678003			82	79
31	155207846	T	C	intronic			0.0002	rs145066479			3	1
32	155207848	G	T	intronic			0.0043	rs183540501			4	2
33	155207866	A	T	intronic				rs529870563			1	2
34	155208167	G	A	intronic				rs566671462			0	2
35	155208495	G	A	intronic			0.0003	rs567935648			0	1
36	155208519	CT	C	intronic			6.673e-05				1	2
37	155208611	A	G	intronic			0.0003	rs569282073			2	1
38	155208624	A	G	intronic			0.0129	rs188328778			15	17
39	155208644	C	T	intronic			6.675e-05				0	1
40	155208647	T	C	intronic			0.7089	rs7416991			687	734
41	155208647	T	G	intronic			0.2911	rs7416991			316	349
42	155208647	T	G	intronic			0.2911	rs7416991			65	72
43	155208805	C	T	intronic			0.0				1	0
44	155208851	T	C	intronic			6.774e-05	rs149120852			0	1
45	155209078	C	T	intronic			0.0	rs1005434278			1	0
46	155209079	G	A	intronic			0.0001	rs114452199			3	2
47	155209082	A	G	intronic				rs899199374			1	2
48	155209251	G	A	intronic				rs991547343			1	0
49	155209297	C	T	intronic			0.0011	rs183903019			4	3
50	155209298	G	A	intronic			0.0002	rs776425625			0	2
51	155209594	G	A	intronic			0.0001	rs377315750			1	0
52	155209913	T	G	intronic			0.0005	rs199565854			0	2
53	155209938	G	A	intronic			0.0003	rs559516544			5	5
54	155209962	C	T	intronic			0.0115	rs114217696			14	12
55	155210030	G	A	intronic			0.0001	rs151028758			3	2
56	155210070	C	CA	intronic							0	1
57	155210146	C	T	intronic			0.0001				2	1
58	155210156	G	C	intronic							0	1
59	155210170	G	A	intronic			0.0002	rs962460364			0	1
60	155210570	T	C	intronic			0.001	rs2361534			0	2
61	155210613	C	T	intronic							1	1
62	155210641	A	C	intronic			0.0002	rs2070679			3	3
63	155210723	C	T	intronic							1	0
64	155210739	C	T	intronic							1	0
65	155210918	T	C	UTR5	NM_001005742	c.-15A>G	0.0013	rs41264927			3	2
66	155211027	C	T	UTR5	NM_000157	c.-124G>A					0	1
67	155211089	C	T	intronic							1	0
68	155211101	G	A	intronic			0.0	rs1007847984			0	1
69	155211106	T	C	intronic			0.0116	rs188978150		Uncertain_significance	30	27

Abbreviations: GD, Gaucher's disease; PD, Parkinson's disease; DLB, Dementia with Lewy Bodies.; gnomAD, The Genome Aggregation Database. DM, Disease causing mutation; FP, in vitro or in vivo functional polymorphism.

Supplementary Table 6.

Type of data	Clinical characteristics and scales	PD *GBA1* carrier Yes (n=67)	PD *GBA1* carrier No (N=554)	missing values (%)	β (95%)	*p*-value	adj *p*-value
Motor symptoms/scales	H&Y, mean (SD)	2.2 (±0.8)	2.2 (±0.8)	3 (0.5%)	0.1 (-0.07 to 0.27)	0.2700	0.9635
	MDS-UPDRS II, mean (SD)	11.4 (±7.3)	11.4 (±8.3)	13 (2.1%)	0.34 (-1.53 to 2.21)	0.7219	0.9701
	MDS-UPDRS III, mean (SD)	33.1 (±16.5)	34.6 (±16.2)	15 (2.4%)	-0.69 (-4.61 to 3.22)	0.7294	0.9701
	MDS-UPDRS IV, mean (SD)	1.6 (±3.2)	1.6 (±3.3)	7 (1.1%)	-0.03 (-0.79 to 0.72)	0.9296	0.9701
	Dyskinesias, n (%)	9 (13.4%)	64 (11.6%)	0	0.23 (-0.58 to 1.05)	0.5747	0.9701
	Falls, n (%)	12 (17.9%)	93 (16.8%)	0	0.19 (-0.51 to 0.9)	0.5950	0.9701
	Gait Disorder, n (%)	37 (55.2%)	307 (55.4%)	0	0.07 (-0.46 to 0.59)	0.8063	0.9701
	FOG, n (%)	15 (22.4%)	123 (22.2%)	0	0.07 (-0.6 to 0.74)	0.8402	0.9701
	Restless leg syndrome, n (%)	10 (14.9%)	46 (8.3%)	0	0.63 (-0.11 to 1.37)	0.0969	0.8721
	Motor fluctuation, n (%)	10 (14.9%)	93 (16.8%)	0	-0.17 (-0.95 to 0.6)	0.6628	0.9701
Non-motor symptoms/scales	BDI, mean (SD)	9.4 (±5.8)	9.9 (±7.1)	30 (4.8%)	-0.52 (-2.27 to 1.23)	0.5614	0.9701
	MDS-UPDRS Part I, mean (SD)	11.1 (±6.8)	10.5 (±7.0)	15 (2.4%)	0.65 (-1.04 to 2.33)	0.4550	0.9635
	PDSS, mean (SD)	103.0 (±22.8)	105.0 (±24.6)	46 (7.4%)	-2.06 (-8.23 to 4.11)	0.5126	0.9701
	SCOPA-AUT, mean (SD)	14.9 (±7.8)	14.9 (±8.1)	33 (5.3%)	0.15 (-1.82 to 2.12)	0.8789	0.9701
	**Sniffin's stick test, mean (SD)**	**7.0 (±3.7)**	**7.8 (±3.6)**	**8 (1.3%)**	**-1.03 (-1.91 to -0.15)**	**0.0210***	**0.7470**
	SAS, mean (SD)	13.9 (±5.8)	14.1 (±5.7)	35 (5.6%)	0.03 (-1.42 to 1.49)	0.9649	0.9701
	MoCA, mean (SD)	24.7 (±4.2)	24.4 (±4.5)	14 (2.3%)	-0.09 (-1.14 to 0.96)	0.8686	0.9701
	Constipation, n (%)	29 (43.3%)	246 (44.4%)	0	-0.01 (-0.54 to 0.51)	0.9577	0.9701
	Dysphagia, n (%)	15 (22.4%)	145 (26.2%)	0	-0.2 (-0.81 to 0.41)	0.5282	0.9701
	Insomnia, n (%)	17 (25.4%)	151 (27.3%)	0	-0.13 (-0.72 to 0.45)	0.6549	0.9701
	Orthostatism, n (%)	24 (35.8%)	163 (29.4%)	0	0.32 (-0.22 to 0.85)	0.249	0.9635
	Urinary incontinence, n (%)	25 (37.3%)	168 (30.3%)	0	0.38 (-0.16 to 0.92)	0.1718	0.8835
	**Hallucinations, n (%)**	**16 (23.9%)**	**83 (15.0%)**	**0**	**0.65 (0.03 to 1.28)**	**0.0415***	**0.7470**
	Excessive daytime sleepiness, n (%)	23 (34.3%)	170 (30.7%)	0	0.22 (-0.33 to 0.77)	0.4302	0.9635
	ICD, n (%)	6 (9.0%)	53 (9.6%)	0	-0.05 (-0.96 to 0.86)	0.9154	0.9701
	Syncope, n (%)	6 (9.0%)	26 (4.7%)	0	0.81 (-0.14 to 1.77)	0.0933	0.8721
	RBDSQ, n (%)	25 (37.3%)	165 (29.8%)	45 (7.2%)	0.43 (-0.14 to 1.0)	0.1401	0.8835
Other clinical	LEDD (mg/day), mean (SD)	543.4 (±442.6)	513.5 (±404.9)	21 (3.4%)	35.55 (-54.38 to 125.49)	0.4385	0.9635
	PDQ-39, mean (SD)	39.5 (±27.3)	39.3 (±26.7)	51 (8.2%)	-0.12 (-6.49 to 6.24)	0.9701	0.9701
	DBS, n (%)	4 (6.0%)	24 (4.3%)	0	0.63 (-0.64 to 1.9)	0.3285	0.9635
Comorbidities	Diabetes, n (%)	8 (11.9%)	55 (9.9%)	0	0.34 (-0.47 to 1.15)	0.4080	0.9635
	Hypercholesterolemia, n (%)	25 (37.3%)	226 (40.8%)	0	-0.11 (-0.64 to 0.43)	0.6959	0.9701
	Cardiovascular disease, n (%)	10 (14.9%)	116 (20.9%)	0	-0.29 (-1.02 to 0.44)	0.4303	0.9635
	Arterial hypertension, n (%)	23 (34.3%)	248 (44.8%)	0	-0.38 (-0.93 to 0.16)	0.1697	0.8835
	Traumatic Brain Injury, n (%)	11 (16.4%)	122 (22.0%)	0	-0.36 (-1.04 to 0.32)	0.2995	0.9635

We consider severe, mild, and risk *GBA1* variants as pathogenic mutations. We used regression models (linear and logistic). Data are given as mean and standard deviation (SD) for continuous clinical outcomes and as percentage for binary clinical outcomes. Models adjusted for sex, age at assessment, and disease duration. Beta (β) regression coefficient are given with the 95% CI. Statistically significant results are highlighted in bold with a * sign (p-value < 0.05). Abbreviation : p-value, unadjusted p-value; adj p-value, corrected for multiple comparisons using FDR adjustment; AAO, age at onset; H&Y, Hoehn & Yahr; MDS-UPDRS, Movement Disorders Society - Unified Parkinson's Disease Rating Scale; FOG, freezing of gait; BDI, Beck Depression Inventory; PDSS, Parkinson's Disease Sleep Scale; SCOPA-AUT, Scales for Outcomes in Parkinson's Disease-Autonomic questionnaire; SAS, Starkstein apathy scale; MoCA, Montreal Cognitive Assessment; ICD, impulse control disorder; RBDSQ, REM Sleep Behavior Disorder Screening Questionnaire; LEDD, L-dopa equivalent daily dose (mg/day); PDQ-39, Parkinson's Disease quality of life Questionnaire; DBS, Presence of treatment by Deep Brain Stimulation.

Supplementary Table 7. Comparison of clinical scores between known severe variant carriers and four probably severe VUS carriers

Type of data	Clinical characteristics and scales	Severe PD$_{GBA1}$ carrier mean (SD) n=21	Probably severe VUS *GBA1* carriers score (Z-score)			
			p.Y61H	p.L213P	p.A215D	p.R434C
	AAO	58.6 (±13.1)	**38 (-1.5)***	56 (-0.2)	68 (0.7)	62 (0.26)
	DD	6.4 (±4.7)	5 (-0.3)	**17 (2.2)***	**1 (-1.1)***	11 (0.9)
Motor symptoms/scales	H&Y	2.4 (±0.8)	2 (-0.5)	**4 (2)***	2 (-0.5)	3 (0.75)
	MDS-UPDRS II	12.6 (±4.4)	14 (0.32)	**26 (3.05)***	**6 (-1.5)***	**17 (1)***
	MDS-UPDRS III	34.8 (±15.7)	35 (0.01)	**81 (2.94)***	**14 (-1.32)***	44 (0.59)
	MDS-UPDRS IV	3.0 (±4.5)	**10 (1.56)***	**9 (1.33)***	1 (-0.44)	0 (-0.67)
Non-motor symptoms/scales	BDI	12.4 (±5.7)	9 (-0.6)	-	7 (-0.95)	16 (0.63)
	MDS-UPDRS Part I	15.0 (±6.5)	9 (-0.92)	**26 (1.69)***	17 (0.31)	**6 (-1.38)***
	PDSS	98.3 (±20.9)	95 (-0.16)	-	82 (-0.78)	**65 (-1.59)***
	SCOPA-AUT	17.1 (±8.0)	**8 (-1.14)***	-	12 (-0.64)	**27 (1.24)***
	Sniffin's stick test	6.4 (±3.6)	6 (-0.11)	7 (0.17)	8 (0.44)	3 (-0.94)
	SAS	15.8 (±5.2)	14 (-0.35)	-	17 (0.23)	19 (0.62)
	MoCA	24.0 (±4.7)	26 (0.43)	**18 (-1.28)***	20 (-0.85)	**18 (-1.28)***
Other clinical outcomes	LEDD (mg/day)	690.5 (±457.9)	**1378 (1.5)***	675 (-0.03)	675 (-0.03)	975 (0.62)
	PDQ-39	52.0 (±26.3)	42 (-0.38)	-	**14 (-1.44)***	48 (-0.15)

We compared the clinical scores obtained from known severe variants carriers with the four carriers of probably severe VUS (p.Y61H, p.L213P, p.A215D, and p.R434C). To calculate the z-score, we use the formula, $z=(X – \mu)/\sigma$; X is the clinical outcome value, μ is the mean and σ is the standard deviation. The z-score indicates the number of standard deviations from the mean. z-score deviated from the mean with more or less than 1 SD were highlighted in bold with (*) sign and red. Abbreviation: AAO, age at onset; H&Y, Hoehn & Yahr; MDS-UPDRS, Movement Disorders Society - Unified Parkinson's Disease Rating Scale; BDI, Beck Depression Inventory; PDSS, Parkinson's Disease Sleep Scale; SCOPA-AUT, Scales for Outcomes in Parkinson's Disease-Autonomic questionnaire; SAS, Starkstein apathy scale; MoCA, Montreal Cognitive Assessment; PDQ-39, Parkinson's Disease quality of life Questionnaire.

Supplementary Table 8. Frequency of *GBA1* variant in European Parkinson's disease population that performed full *GBA1* gene sequencing.

Population	PD (n)	Screening method	*GBA1* carrier frequency (%)	E365K	T408M	L483P	N409S	RecNcil	Other
**Ashkenazi Jews[10]**	735	Targeted NGS	18	1.6	0	0.3	11.8	0	4.2
**Netherland[11]**	3402	Long-range PCR	15	6.7	2.5	0.6	0.9	0	4.3
**Italy[12]**	874	Complete exon Sanger sequencing	14.3	1.7	0.6	2.3	3.3	0.8	5.3
**Luxembourg (this cohort)**	**644**	**PacBio**	**12**	**3.6**	**2.6**	**1.7**	**1.1**	**0.6**	**3**
**Southern Spain[13]**	532	High-resolution melting analysis (HRM)	11.7	3	0.9	2.4	0.9	0	4.3
**New Zealand[14]**	229	PCR amplicon + nanopore	9.2	4.8	3.1	0	0.4	0	1.3
**Ireland[15]**	314	Complete exon Sanger sequencing	8.3	4.1	1.9	0	0.9	0.9	0.5
**Portugal[16]**	230	X	8.3	0.9	0.9	1.3	2.2	0	0.9
**Greece[17]**	172	X	6.4	0.6	0	1.2	0	0	4.6

Supplementary Table 9. MultiQC report quality control statistics for PacBio data.

Sample N°	total_reads	mapped_reads	general_error_rate	mean_coverage	percentage_aligned
**1**	706	706	0	698,1	100,0
**2**	1059	1059	0	1038,1	100,0
**3**	290	290	0	250,3	100,0
**4**	775	775	0	769,0	100,0
**5**	884	884	0	873,7	100,0
**6**	965	965	0	938,7	100,0
**7**	798	798	0	783,8	100,0
**8**	822	822	0	809,9	100,0
**9**	852	852	0	839,2	100,0
**10**	959	959	0	944,5	100,0
**11**	686	686	0	678,4	100,0
**12**	645	645	0	637,5	100,0
**13**	1037	1037	0	1004,6	100,0
**14**	701	701	0	688,1	100,0
**15**	508	508	0	492,3	100,0
**16**	621	621	0	605,6	100,0
**17**	868	868	0	838,8	100,0
**18**	575	575	0	563,7	100,0
**19**	276	276	0	264,5	100,0
**20**	685	685	0	665,0	100,0
**21**	822	822	0	796,8	100,0
**22**	591	591	0	584,7	100,0
**23**	209	209	0	202,3	100,0
**24**	924	924	0	911,0	100,0
**25**	782	782	0	745,8	100,0
**26**	526	526	0	512,4	100,0
**27**	821	821	0	805,7	100,0
**28**	591	591	0	581,5	100,0
**29**	789	789	0	762,8	100,0
**30**	546	546	0	538,3	100,0
**31**	459	459	0	455,1	100,0
**32**	561	561	0	552,8	100,0
**33**	641	641	0	625,4	100,0
**34**	745	744	0	739,8	99,9
**35**	769	769	0	759,2	100,0
**36**	857	857	0	842,1	100,0
**37**	972	972	0	939,7	100,0
**38**	730	730	0	717,4	100,0
**39**	742	742	0	724,0	100,0
**40**	775	774	0	766,1	99,9
**41**	774	774	0	748,5	100,0
**42**	744	744	0	729,4	100,0
**43**	963	963	0	950,4	100,0
**44**	594	594	0	586,2	100,0
**45**	545	545	0	536,5	100,0
**46**	893	892	0	882,1	99,9
**47**	900	900	0	890,2	100,0
**48**	1036	1036	0	1024,8	100,0
**49**	873	873	0	859,2	100,0
**50**	900	900	0	887,8	100,0
**51**	811	811	0	801,2	100,0
**52**	1070	1069	0	1046,8	99,9
**53**	634	634	0	621,2	100,0
**54**	999	999	0	995,2	100,0
**55**	761	761	0	748,6	100,0
**56**	1122	1122	0	1108,9	100,0
**57**	1008	1008	0	993,8	100,0
**58**	988	988	0	973,5	100,0
**59**	1078	1078	0	1042,6	100,0
**60**	1354	1354	0	1317,1	100,0
**61**	826	826	0	802,1	100,0
**62**	641	641	0	628,9	100,0
**63**	746	746	0	721,9	100,0
**64**	1051	1051	0	1015,2	100,0
**65**	702	702	0	687,1	100,0
**66**	446	446	0	432,7	100,0
**67**	907	907	0	892,2	100,0
**68**	1108	1108	0	1087,7	100,0
**69**	618	618	0	614,0	100,0
**70**	732	732	0	725,7	100,0
**71**	1134	1134	0	1119,9	100,0
**72**	767	767	0	743,9	100,0
**73**	795	795	0	781,6	100,0
**74**	551	551	0	541,9	100,0
**75**	808	808	0	805,0	100,0
**76**	895	895	0	880,9	100,0
**77**	811	811	0	800,5	100,0
**78**	731	731	0	724,5	100,0
**79**	979	979	0	970,8	100,0
**80**	972	972	0	965,1	100,0
**81**	668	668	0	660,6	100,0
**82**	981	981	0	967,9	100,0
**83**	769	769	0	750,7	100,0
**84**	606	606	0	597,6	100,0
**85**	474	474	0	468,9	100,0
**86**	620	620	0	612,9	100,0
**87**	861	861	0	850,3	100,0
**88**	783	783	0	760,0	100,0
**89**	751	751	0	746,9	100,0
**90**	259	259	0	248,4	100,0
**91**	897	897	0	866,9	100,0
**92**	988	988	0	976,7	100,0
**93**	673	673	0	655,9	100,0
**94**	1174	1174	0	1159,7	100,0
**95**	944	944	0	930,6	100,0
**96**	873	873	0	854,9	100,0
**97**	590	590	0	566,9	100,0
**98**	785	785	0	772,6	100,0
**99**	743	743	0	712,8	100,0
**100**	621	621	0	614,9	100,0

**101**	639	639	0	614,2	100,0
**102**	736	736	0	705,1	100,0
**103**	543	543	0	521,1	100,0
**104**	524	524	0	511,7	100,0
**105**	601	601	0	594,7	100,0
**106**	402	402	0	384,4	100,0
**107**	684	684	0	676,3	100,0
**108**	591	590	0	572,1	99,8
**109**	629	629	0	623,2	100,0
**110**	849	849	0	831,4	100,0
**111**	803	803	0	795,7	100,0
**112**	553	553	0	541,6	100,0
**113**	788	788	0	762,6	100,0
**114**	962	962	0	953,6	100,0
**115**	606	606	0	576,0	100,0
**116**	939	939	0	924,6	100,0
**117**	707	707	0	700,2	100,0
**118**	773	773	0	762,6	100,0
**119**	710	709	0	688,7	99,9
**120**	557	557	0	542,1	100,0
**121**	679	679	0	673,1	100,0
**122**	951	951	0	916,7	100,0
**123**	855	855	0	847,0	100,0
**124**	709	709	0	698,8	100,0
**125**	884	884	0	857,1	100,0
**126**	829	829	0	814,3	100,0
**127**	781	781	0	761,7	100,0
**128**	1044	1044	0	1029,3	100,0
**129**	858	858	0	850,0	100,0
**130**	1015	1014	0	1004,6	99,9
**131**	871	871	0	864,5	100,0
**132**	752	752	0	748,1	100,0
**133**	1016	1016	0	1011,7	100,0
**134**	1128	1128	0	1098,0	100,0
**135**	987	987	0	985,9	100,0
**136**	282	282	0	273,8	100,0
**137**	1179	1179	0	1139,1	100,0
**138**	1061	1061	0	1053,2	100,0
**139**	1007	1006	0	987,2	99,9
**140**	1251	1250	0	1242,2	99,9
**141**	1180	1180	0	1176,9	100,0
**142**	1201	1200	0	1195,4	99,9
**143**	1032	1030	0	1013,7	99,8
**144**	740	740	0	717,5	100,0
**145**	1020	1020	0	1008,3	100,0
**146**	1233	1233	0	1213,1	100,0
**147**	1102	1102	0	1089,1	100,0
**148**	1079	1079	0	1041,1	100,0
**149**	801	801	0	781,6	100,0
**150**	907	907	0	886,7	100,0
**151**	1445	1445	0	1422,4	100,0
**152**	835	835	0	821,7	100,0
**153**	575	575	0	560,7	100,0
**154**	1235	1235	0	1213,1	100,0
**155**	692	692	0	657,9	100,0
**156**	1078	1078	0	1066,9	100,0
**157**	807	807	0	797,9	100,0
**158**	1067	1067	0	1057,0	100,0
**159**	832	832	0	824,1	100,0
**160**	1096	1096	0	1073,9	100,0
**161**	825	825	0	798,9	100,0
**162**	1296	1296	0	1278,8	100,0
**163**	876	876	0	870,9	100,0
**164**	629	629	0	623,3	100,0
**165**	946	946	0	935,5	100,0
**166**	782	782	0	764,3	100,0
**167**	737	737	0	729,5	100,0
**168**	918	918	0	899,3	100,0
**169**	840	840	0	837,8	100,0
**170**	757	757	0	747,0	100,0
**171**	863	863	0	832,5	100,0
**172**	853	853	0	843,3	100,0
**173**	672	672	0	658,8	100,0
**174**	851	851	0	838,8	100,0
**175**	897	897	0	890,7	100,0
**176**	820	820	0	807,0	100,0
**177**	1238	1238	0	1234,7	100,0
**178**	642	642	0	618,0	100,0
**179**	1113	1113	0	1096,3	100,0
**180**	1196	1196	0	1179,7	100,0
**181**	729	729	0	724,2	100,0
**182**	1095	1095	0	1087,5	100,0
**183**	1317	1317	0	1306,0	100,0
**184**	1119	1119	0	1107,5	100,0
**185**	1730	1730	0	1720,2	100,0
**186**	873	873	0	852,3	100,0
**187**	1019	1019	0	999,2	100,0
**188**	956	956	0	941,6	100,0
**189**	859	859	0	855,9	100,0
**190**	1384	1383	0	1372,8	99,9
**191**	1488	1488	0	1475,7	100,0
**192**	1071	1070	0	1056,3	99,9
**193**	818	818	0	801,2	100,0
**194**	1183	1183	0	1173,4	100,0
**195**	1096	1096	0	1084,2	100,0
**196**	765	765	0	759,8	100,0
**197**	605	605	0	599,8	100,0
**198**	927	927	0	922,8	100,0
**199**	970	969	0	963,0	99,9
**200**	973	973	0	964,4	100,0
**201**	1235	1235	0	1225,5	100,0
**202**	1480	1480	0	1460,4	100,0
**203**	2528	2528	0	2518,9	100,0
**204**	952	952	0	931,0	100,0
**205**	1228	1228	0	1215,0	100,0

**206**	1242	1242	0	1217,3	100,0
**207**	931	931	0	929,7	100,0
**208**	1271	1271	0	1263,8	100,0
**209**	1473	1473	0	1463,2	100,0
**210**	1266	1266	0	1254,4	100,0
**211**	700	700	0	681,4	100,0
**212**	922	922	0	900,0	100,0
**213**	966	966	0	963,5	100,0
**214**	1118	1118	0	1113,6	100,0
**215**	1534	1534	0	1527,4	100,0
**216**	1369	1368	0	1357,3	99,9
**217**	1726	1726	0	1703,7	100,0
**218**	988	988	0	951,5	100,0
**219**	1102	1101	0	1086,3	99,9
**220**	776	776	0	767,5	100,0
**221**	1281	1281	0	1272,5	100,0
**222**	1409	1409	0	1393,1	100,0
**223**	2040	2040	0	2022,6	100,0
**224**	415	415	0	407,5	100,0
**225**	1028	1028	0	1008,6	100,0
**226**	1257	1257	0	1246,8	100,0
**227**	1205	1205	0	1198,1	100,0
**228**	1143	1143	0	1132,8	100,0
**229**	804	804	0	788,2	100,0
**230**	1056	1056	0	1048,8	100,0
**231**	1192	1192	0	1179,2	100,0
**232**	1150	1149	0	1144,5	99,9
**233**	995	995	0	970,0	100,0
**234**	919	918	0	913,2	99,9
**235**	209	209	0	189,8	100,0
**236**	861	861	0	857,0	100,0
**237**	867	867	0	863,6	100,0
**238**	1035	1035	0	1014,9	100,0
**239**	1671	1670	0	1660,7	99,9
**240**	934	934	0	931,3	100,0
**241**	767	767	0	756,9	100,0
**242**	923	923	0	909,5	100,0
**243**	796	796	0	770,0	100,0
**244**	719	719	0	716,9	100,0
**245**	449	449	0	438,0	100,0
**246**	760	760	0	736,9	100,0
**247**	786	786	0	777,2	100,0
**248**	635	635	0	625,5	100,0
**249**	268	268	0	262,9	100,0
**250**	729	729	0	719,4	100,0
**251**	848	848	0	835,0	100,0
**252**	706	706	0	683,2	100,0
**253**	706	706	0	697,8	100,0
**254**	773	773	0	765,3	100,0
**255**	787	787	0	774,9	100,0
**256**	794	794	0	780,0	100,0
**257**	724	724	0	719,1	100,0
**258**	591	591	0	586,8	100,0
**259**	870	870	0	858,3	100,0
**260**	749	749	0	740,0	100,0
**261**	1027	1027	0	1018,1	100,0
**262**	1136	1136	0	1102,4	100,0
**263**	1125	1125	0	1101,1	100,0
**264**	821	821	0	819,1	100,0
**265**	873	873	0	869,1	100,0
**266**	1083	1083	0	1066,0	100,0
**267**	966	966	0	962,1	100,0
**268**	884	884	0	866,4	100,0
**269**	710	710	0	697,6	100,0
**270**	1234	1234	0	1222,0	100,0
**271**	1217	1217	0	1192,3	100,0
**272**	394	394	0	367,9	100,0
**273**	994	994	0	988,0	100,0
**274**	1044	1044	0	1036,4	100,0
**275**	1273	1273	0	1260,9	100,0
**276**	1133	1133	0	1120,3	100,0
**277**	938	938	0	934,3	100,0
**278**	1065	1065	0	1059,3	100,0
**279**	1073	1073	0	1057,4	100,0
**280**	888	888	0	876,3	100,0
**281**	1337	1337	0	1312,7	100,0
**282**	1110	1110	0	1093,5	100,0
**283**	1159	1159	0	1145,1	100,0
**284**	1319	1319	0	1284,2	100,0
**285**	1046	1046	0	1035,0	100,0
**286**	724	724	0	712,2	100,0
**287**	1276	1276	0	1250,6	100,0
**288**	1124	1124	0	1096,1	100,0
**289**	858	858	0	840,5	100,0
**290**	450	450	0	441,0	100,0
**291**	1438	1438	0	1419,3	100,0
**292**	1037	1037	0	1011,5	100,0
**293**	931	931	0	912,8	100,0
**294**	1083	1083	0	1077,7	100,0
**295**	1096	1096	0	1085,0	100,0
**296**	1139	1139	0	1121,6	100,0
**297**	965	965	0	958,7	100,0
**298**	773	773	0	770,0	100,0
**299**	1200	1199	0	1179,8	99,9
**300**	960	960	0	952,2	100,0
**301**	877	877	0	863,6	100,0
**302**	1144	1144	0	1136,0	100,0
**303**	1002	1002	0	995,6	100,0
**304**	574	574	0	556,6	100,0
**305**	875	875	0	873,3	100,0
**306**	795	795	0	791,9	100,0
**307**	841	841	0	829,2	100,0
**308**	726	726	0	719,1	100,0
**309**	822	822	0	814,9	100,0
**310**	667	667	0	659,9	100,0

**311**	1183	1183	0	1171,0	100,0
**312**	862	862	0	842,8	100,0
**313**	859	859	0	846,6	100,0
**314**	432	432	0	424,6	100,0
**315**	969	969	0	961,2	100,0
**316**	1278	1277	0	1258,7	99,9
**317**	724	723	0	716,0	99,9
**318**	1036	1036	0	1032,0	100,0
**319**	811	811	0	802,1	100,0
**320**	1476	1476	0	1461,6	100,0
**321**	1193	1193	0	1188,4	100,0
**322**	1651	1651	0	1625,7	100,0
**323**	1352	1352	0	1333,6	100,0
**324**	1634	1634	0	1608,0	100,0
**325**	1061	1061	0	1043,7	100,0
**326**	1027	1027	0	1016,2	100,0
**327**	1186	1186	0	1167,4	100,0
**328**	1617	1617	0	1604,7	100,0
**329**	1683	1683	0	1673,6	100,0
**330**	738	738	0	734,4	100,0
**331**	1931	1931	0	1923,8	100,0
**332**	1954	1954	0	1911,0	100,0
**333**	1361	1361	0	1351,3	100,0
**334**	1378	1378	0	1373,5	100,0
**335**	1872	1872	0	1856,1	100,0
**336**	1041	1041	0	1027,0	100,0
**337**	1139	1139	0	1132,3	100,0
**338**	1170	1170	0	1161,7	100,0
**339**	1769	1768	0	1756,3	99,9
**340**	1556	1556	0	1523,0	100,0
**341**	1221	1221	0	1209,2	100,0
**342**	1061	1061	0	1047,4	100,0
**343**	1296	1295	0	1277,4	99,9
**344**	955	954	0	946,6	99,9
**345**	1233	1233	0	1230,1	100,0
**346**	864	864	0	852,5	100,0
**347**	565	565	0	560,4	100,0
**348**	598	598	0	590,4	100,0
**349**	589	589	0	588,3	100,0
**350**	792	792	0	782,8	100,0
**351**	250	250	0	228,3	100,0
**352**	372	372	0	361,3	100,0
**353**	637	637	0	628,3	100,0
**354**	724	724	0	703,7	100,0
**355**	526	526	0	515,2	100,0
**356**	597	596	0	594,4	99,8
**357**	502	502	0	491,4	100,0
**358**	670	670	0	663,5	100,0
**359**	399	399	0	393,0	100,0
**360**	304	304	0	301,5	100,0
**361**	709	709	0	706,4	100,0
**362**	804	803	0	779,1	99,9
**363**	516	516	0	509,0	100,0
**364**	540	540	0	531,9	100,0
**365**	755	755	0	740,5	100,0
**366**	429	429	0	418,8	100,0
**367**	308	308	0	303,2	100,0
**368**	583	583	0	577,1	100,0
**369**	616	616	0	605,8	100,0
**370**	586	586	0	580,3	100,0
**371**	681	681	0	670,6	100,0
**372**	346	346	0	342,2	100,0
**373**	846	846	0	823,9	100,0
**374**	540	540	0	535,7	100,0
**375**	588	588	0	585,7	100,0
**376**	769	769	0	750,3	100,0
**377**	448	448	0	439,8	100,0
**378**	546	546	0	542,4	100,0
**379**	622	622	0	615,3	100,0
**380**	823	822	0	815,1	99,9
**381**	710	710	0	706,9	100,0
**382**	598	598	0	593,7	100,0
**383**	775	775	0	765,1	100,0
**384**	964	964	0	926,0	100,0
**385**	652	652	0	639,0	100,0
**386**	566	566	0	559,5	100,0
**387**	785	785	0	761,5	100,0
**388**	517	517	0	510,5	100,0
**389**	574	574	0	567,9	100,0
**390**	341	341	0	335,9	100,0
**391**	773	773	0	750,1	100,0
**392**	906	906	0	896,4	100,0
**393**	702	702	0	680,4	100,0
**394**	958	958	0	946,0	100,0
**395**	661	661	0	657,5	100,0
**396**	772	772	0	764,8	100,0
**397**	736	736	0	726,6	100,0
**398**	585	585	0	559,4	100,0
**399**	743	742	0	730,6	99,9
**400**	1070	1070	0	1050,8	100,0
**401**	885	885	0	850,5	100,0
**402**	599	599	0	587,0	100,0
**403**	858	858	0	837,1	100,0
**404**	1077	1076	0	1054,4	99,9
**405**	693	693	0	682,6	100,0
**406**	500	500	0	484,4	100,0
**407**	763	763	0	739,6	100,0
**408**	749	749	0	741,1	100,0
**409**	1131	1131	0	1103,4	100,0
**410**	616	616	0	612,5	100,0
**411**	991	991	0	986,5	100,0
**412**	1025	1025	0	983,6	100,0
**413**	892	892	0	876,3	100,0
**414**	1292	1291	0	1274,3	99,9
**415**	1125	1125	0	1118,8	100,0

**416**	927	927	0	916,7	100,0
**417**	1126	1126	0	1117,3	100,0
**418**	663	663	0	638,9	100,0
**419**	590	590	0	584,8	100,0
**420**	613	613	0	587,2	100,0
**421**	585	585	0	581,5	100,0
**422**	801	801	0	796,4	100,0
**423**	900	900	0	873,8	100,0
**424**	900	900	0	892,5	100,0
**425**	616	616	0	603,3	100,0
**426**	777	777	0	771,7	100,0
**427**	879	879	0	877,5	100,0
**428**	932	931	0	922,4	99,9
**429**	962	962	0	956,7	100,0
**430**	1060	1060	0	1048,1	100,0
**431**	778	778	0	772,9	100,0
**432**	760	760	0	741,1	100,0
**433**	651	651	0	644,7	100,0
**434**	218	218	0	212,4	100,0
**435**	1107	1106	0	1082,7	99,9
**436**	1201	1201	0	1191,1	100,0
**437**	847	847	0	831,6	100,0
**438**	937	937	0	928,1	100,0
**439**	1013	1013	0	1003,4	100,0
**440**	1079	1079	0	1070,4	100,0
**441**	691	691	0	668,9	100,0
**442**	650	650	0	645,9	100,0
**443**	762	762	0	741,1	100,0
**444**	901	900	0	899,1	99,9
**445**	847	847	0	838,9	100,0
**446**	808	808	0	785,6	100,0
**447**	771	771	0	765,3	100,0
**448**	665	665	0	646,7	100,0
**449**	962	962	0	954,4	100,0
**450**	883	883	0	881,6	100,0
**451**	417	417	0	409,7	100,0
**452**	765	765	0	756,7	100,0
**453**	546	546	0	525,8	100,0
**454**	640	640	0	636,3	100,0
**455**	758	758	0	734,4	100,0
**456**	768	768	0	764,6	100,0
**457**	873	873	0	869,4	100,0
**458**	1053	1053	0	1031,5	100,0
**459**	1035	1035	0	1022,9	100,0
**460**	1120	1120	0	1113,8	100,0
**461**	931	931	0	925,3	100,0
**462**	788	788	0	774,0	100,0
**463**	637	637	0	630,4	100,0
**464**	796	796	0	783,3	100,0
**465**	802	802	0	793,7	100,0
**466**	957	957	0	949,1	100,0
**467**	1046	1046	0	1022,6	100,0
**468**	1260	1260	0	1248,1	100,0
**469**	881	881	0	856,4	100,0
**470**	1069	1069	0	1056,6	100,0
**471**	1014	1014	0	1011,4	100,0
**472**	741	741	0	734,3	100,0
**473**	513	513	0	507,9	100,0
**474**	282	282	0	267,9	100,0
**475**	822	822	0	814,1	100,0
**476**	834	834	0	825,8	100,0
**477**	751	751	0	745,0	100,0
**478**	127	127	0	124,6	100,0
**479**	766	766	0	761,9	100,0
**480**	622	622	0	619,0	100,0
**481**	873	873	0	865,7	100,0
**482**	999	999	0	988,5	100,0
**483**	711	711	0	694,4	100,0
**484**	871	871	0	861,2	100,0
**485**	682	682	0	678,8	100,0
**486**	813	813	0	793,1	100,0
**487**	1020	1020	0	1005,9	100,0
**488**	632	632	0	616,9	100,0
**489**	749	749	0	738,2	100,0
**490**	632	632	0	628,9	100,0
**491**	430	430	0	423,0	100,0
**492**	1141	1141	0	1119,6	100,0
**493**	1186	1186	0	1176,3	100,0
**494**	618	618	0	599,2	100,0
**495**	930	930	0	918,3	100,0
**496**	868	868	0	866,5	100,0
**497**	974	974	0	961,1	100,0
**498**	923	923	0	902,4	100,0
**499**	813	813	0	806,4	100,0
**500**	704	704	0	703,2	100,0
**501**	1022	1022	0	1015,9	100,0
**502**	673	673	0	668,6	100,0
**503**	466	466	0	456,0	100,0
**504**	917	917	0	910,1	100,0
**505**	742	742	0	738,0	100,0
**506**	905	905	0	904,4	100,0
**507**	576	576	0	570,3	100,0
**508**	639	639	0	617,9	100,0
**509**	834	834	0	826,3	100,0
**510**	815	814	0	800,4	99,9
**511**	588	588	0	583,7	100,0
**512**	435	435	0	429,9	100,0
**513**	617	617	0	615,7	100,0
**514**	887	887	0	884,6	100,0
**515**	661	661	0	652,5	100,0
**516**	1032	1032	0	1029,4	100,0
**517**	710	710	0	701,7	100,0
**518**	1135	1135	0	1130,5	100,0
**519**	723	723	0	718,2	100,0
**520**	188	188	0	168,1	100,0

**521**	673	673	0	659,7	100,0
**522**	893	893	0	886,2	100,0
**523**	858	858	0	850,5	100,0
**524**	261	261	0	260,2	100,0
**525**	388	388	0	381,9	100,0
**526**	1031	1031	0	1025,2	100,0
**527**	972	972	0	956,1	100,0
**528**	874	874	0	856,7	100,0
**529**	762	762	0	755,4	100,0
**530**	548	548	0	537,7	100,0
**531**	658	658	0	654,8	100,0
**532**	332	332	0	332,0	100,0
**533**	639	639	0	638,2	100,0
**534**	530	530	0	527,9	100,0
**535**	1007	1007	0	1001,3	100,0
**536**	980	980	0	972,6	100,0
**537**	741	741	0	736,0	100,0
**538**	805	805	0	780,3	100,0
**539**	989	989	0	984,5	100,0
**540**	1052	1052	0	1026,3	100,0
**541**	754	754	0	748,6	100,0
**542**	920	920	0	917,3	100,0
**543**	1283	1283	0	1272,2	100,0
**544**	1074	1074	0	1065,6	100,0
**545**	1190	1190	0	1183,4	100,0
**546**	1059	1059	0	1054,8	100,0
**547**	630	630	0	611,6	100,0
**548**	1354	1353	0	1338,6	99,9
**549**	1088	1088	0	1070,2	100,0
**550**	782	782	0	777,4	100,0
**551**	667	667	0	663,4	100,0
**552**	1041	1041	0	1032,4	100,0
**553**	807	807	0	796,4	100,0
**554**	703	703	0	695,4	100,0
**555**	186	186	0	170,6	100,0
**556**	816	816	0	809,0	100,0
**557**	1061	1061	0	1053,3	100,0
**558**	715	715	0	702,5	100,0
**559**	889	889	0	885,9	100,0
**560**	799	799	0	794,3	100,0
**561**	829	829	0	818,9	100,0
**562**	923	923	0	910,2	100,0
**563**	926	926	0	911,5	100,0
**564**	658	658	0	647,5	100,0
**565**	917	917	0	907,4	100,0
**566**	947	947	0	922,3	100,0
**567**	974	974	0	963,5	100,0
**568**	604	604	0	596,0	100,0
**569**	1047	1047	0	1025,8	100,0
**570**	1004	1004	0	989,3	100,0
**571**	726	726	0	718,6	100,0
**572**	562	561	0	553,3	99,8
**573**	1027	1027	0	1009,9	100,0
**574**	876	876	0	865,5	100,0
**575**	1042	1042	0	1034,5	100,0
**576**	860	860	0	844,4	100,0
**577**	1038	1038	0	1033,1	100,0
**578**	1088	1088	0	1054,9	100,0
**579**	991	991	0	983,9	100,0
**580**	890	890	0	887,6	100,0
**581**	1262	1262	0	1245,3	100,0
**582**	1153	1153	0	1143,2	100,0
**583**	883	883	0	877,9	100,0
**584**	820	820	0	815,3	100,0
**585**	931	931	0	924,0	100,0
**586**	1072	1072	0	1063,9	100,0
**587**	779	779	0	770,8	100,0
**588**	794	794	0	774,5	100,0
**589**	929	929	0	922,6	100,0
**590**	852	852	0	840,8	100,0
**591**	695	695	0	691,1	100,0
**592**	690	690	0	684,8	100,0
**593**	1022	1022	0	1011,0	100,0
**594**	947	947	0	937,6	100,0
**595**	1161	1161	0	1145,0	100,0
**596**	892	892	0	889,9	100,0
**597**	489	489	0	469,4	100,0
**598**	1091	1091	0	1086,2	100,0
**599**	1080	1080	0	1076,7	100,0
**600**	1309	1309	0	1299,0	100,0
**601**	1070	1070	0	1061,1	100,0
**602**	927	927	0	925,7	100,0
**603**	988	988	0	984,3	100,0
**604**	1023	1023	0	1014,1	100,0
**605**	829	829	0	823,8	100,0
**606**	947	947	0	942,0	100,0
**607**	807	807	0	799,6	100,0
**608**	881	881	0	877,5	100,0
**609**	994	994	0	971,5	100,0
**610**	901	901	0	895,2	100,0
**611**	499	499	0	495,8	100,0
**612**	807	807	0	798,4	100,0
**613**	699	699	0	696,5	100,0
**614**	320	320	0	317,0	100,0
**615**	906	906	0	898,1	100,0
**616**	959	959	0	951,5	100,0
**617**	1118	1118	0	1113,2	100,0
**618**	1038	1038	0	1028,9	100,0
**619**	1166	1166	0	1159,6	100,0
**620**	1082	1082	0	1060,5	100,0
**621**	1047	1047	0	1045,5	100,0
**622**	1098	1098	0	1096,2	100,0
**623**	1141	1141	0	1125,5	100,0
**624**	928	928	0	918,5	100,0
**625**	1038	1038	0	1035,7	100,0

**626**	925	925	0	920,1	100,0
**627**	1160	1160	0	1154,4	100,0
**628**	1016	1016	0	1003,1	100,0
**629**	1025	1025	0	1018,2	100,0
**630**	1091	1091	0	1071,3	100,0
**631**	1270	1270	0	1266,4	100,0
**632**	1214	1214	0	1187,7	100,0
**633**	1063	1062	0	1056,4	99,9
**634**	1135	1135	0	1128,3	100,0
**635**	1183	1183	0	1174,2	100,0
**636**	941	941	0	929,5	100,0
**637**	1332	1332	0	1326,9	100,0
**638**	739	739	0	731,9	100,0
**639**	927	927	0	915,6	100,0
**640**	431	431	0	410,1	100,0
**641**	1012	1012	0	999,8	100,0
**642**	987	987	0	977,4	100,0
**643**	1124	1123	0	1106,8	99,9
**644**	774	774	0	766,6	100,0
**645**	957	956	0	953,4	99,9
**646**	1192	1192	0	1177,9	100,0
**647**	891	891	0	882,4	100,0
**648**	727	727	0	722,4	100,0
**649**	946	946	0	928,1	100,0
**650**	1162	1162	0	1137,9	100,0
**651**	818	818	0	809,0	100,0
**652**	597	597	0	584,0	100,0
**653**	924	924	0	909,3	100,0
**654**	1078	1078	0	1057,8	100,0
**655**	751	751	0	744,3	100,0
**656**	976	976	0	971,4	100,0
**657**	936	935	0	920,6	99,9
**658**	696	695	0	684,6	99,9
**659**	464	464	0	450,6	100,0
**660**	819	819	0	810,1	100,0
**661**	785	785	0	762,9	100,0
**662**	699	698	0	687,6	99,9
**663**	755	755	0	748,7	100,0
**664**	664	664	0	661,8	100,0
**665**	941	941	0	925,5	100,0
**666**	563	563	0	556,7	100,0
**667**	668	668	0	660,3	100,0
**668**	886	886	0	881,1	100,0
**669**	737	737	0	729,8	100,0
**670**	654	654	0	647,6	100,0
**671**	915	915	0	905,6	100,0
**672**	925	925	0	910,5	100,0
**673**	807	807	0	801,1	100,0
**674**	860	860	0	856,9	100,0
**675**	795	795	0	788,6	100,0
**676**	951	951	0	939,3	100,0
**677**	853	853	0	846,2	100,0
**678**	984	984	0	981,4	100,0
**679**	797	797	0	793,6	100,0
**680**	741	741	0	734,6	100,0
**681**	880	880	0	878,1	100,0
**682**	1080	1078	0	1076,4	99,8
**683**	1051	1051	0	1042,8	100,0
**684**	353	353	0	340,4	100,0
**685**	1227	1227	0	1220,9	100,0
**686**	1157	1157	0	1146,9	100,0
**687**	1189	1189	0	1172,9	100,0
**688**	928	928	0	918,1	100,0
**689**	1206	1206	0	1197,6	100,0
**690**	420	420	0	415,2	100,0
**691**	1345	1345	0	1333,5	100,0
**692**	873	873	0	865,9	100,0
**693**	532	532	0	524,3	100,0
**694**	1107	1107	0	1096,9	100,0
**695**	1226	1226	0	1206,0	100,0
**696**	939	939	0	930,6	100,0
**697**	687	687	0	681,7	100,0
**698**	864	864	0	853,3	100,0
**699**	1103	1103	0	1090,1	100,0
**700**	665	665	0	654,1	100,0
**701**	540	540	0	534,5	100,0
**702**	773	773	0	765,2	100,0
**703**	1192	1192	0	1180,5	100,0
**704**	1019	1019	0	1006,9	100,0
**705**	885	885	0	871,9	100,0
**706**	1000	1000	0	981,4	100,0
**707**	1501	1501	0	1473,1	100,0
**708**	1099	1099	0	1091,1	100,0
**709**	854	854	0	841,7	100,0
**710**	1435	1434	0	1423,3	99,9
**711**	706	706	0	699,8	100,0
**712**	944	944	0	940,0	100,0
**713**	1076	1076	0	1069,5	100,0
**714**	786	786	0	776,0	100,0
**715**	749	749	0	745,6	100,0
**716**	1130	1130	0	1115,0	100,0
**717**	1373	1373	0	1351,7	100,0
**718**	1108	1108	0	1091,4	100,0
**719**	635	635	0	627,8	100,0
**720**	1031	1030	0	1011,1	99,9
**721**	706	706	0	697,3	100,0
**722**	414	414	0	409,6	100,0
**723**	1076	1075	0	1057,7	99,9
**724**	983	983	0	974,3	100,0
**725**	1014	1014	0	1007,4	100,0
**726**	1105	1105	0	1092,6	100,0
**727**	1138	1138	0	1129,1	100,0
**728**	764	764	0	753,3	100,0
**729**	1075	1075	0	1066,3	100,0
**730**	992	991	0	965,2	99,9

**731**	1099	1099	0	1094,9	100,0
**732**	566	566	0	541,5	100,0
**733**	788	788	0	768,8	100,0
**734**	856	856	0	850,9	100,0
**735**	1254	1254	0	1244,0	100,0
**736**	957	957	0	952,6	100,0
**737**	658	658	0	650,6	100,0
**738**	889	889	0	880,3	100,0
**739**	739	739	0	721,4	100,0
**740**	792	792	0	781,6	100,0
**741**	687	687	0	683,9	100,0
**742**	990	990	0	978,8	100,0
**743**	740	740	0	721,9	100,0
**744**	885	885	0	877,6	100,0
**745**	726	726	0	720,2	100,0
**746**	808	808	0	803,0	100,0
**747**	688	688	0	681,6	100,0
**748**	547	547	0	534,6	100,0
**749**	891	891	0	876,2	100,0
**750**	492	492	0	483,4	100,0
**751**	890	890	0	885,3	100,0
**752**	1128	1128	0	1120,9	100,0
**753**	1154	1154	0	1148,8	100,0
**754**	1170	1170	0	1160,0	100,0
**755**	948	948	0	945,7	100,0
**756**	154	154	0	152,1	100,0
**757**	1142	1142	0	1119,8	100,0
**758**	1277	1277	0	1272,8	100,0
**759**	995	995	0	984,4	100,0
**760**	1258	1258	0	1255,7	100,0
**761**	1204	1204	0	1193,6	100,0
**762**	1131	1130	0	1122,0	99,9
**763**	1253	1253	0	1244,3	100,0
**764**	1055	1054	0	1037,5	99,9
**765**	943	943	0	924,6	100,0
**766**	1145	1145	0	1140,9	100,0
**767**	1051	1051	0	1037,8	100,0
**768**	1058	1058	0	1042,4	100,0
**769**	654	654	0	646,4	100,0
**770**	785	785	0	767,1	100,0
**771**	1231	1231	0	1224,9	100,0
**772**	724	724	0	723,2	100,0
**773**	547	547	0	541,4	100,0
**774**	1085	1085	0	1075,5	100,0
**775**	817	817	0	803,9	100,0
**776**	1323	1323	0	1310,9	100,0
**777**	997	997	0	988,9	100,0
**778**	1092	1092	0	1072,1	100,0
**779**	1083	1083	0	1072,5	100,0
**780**	871	871	0	859,9	100,0
**781**	1380	1380	0	1364,3	100,0
**782**	966	966	0	961,0	100,0
**783**	835	835	0	823,1	100,0
**784**	633	633	0	619,7	100,0
**785**	825	825	0	809,2	100,0
**786**	971	971	0	963,6	100,0
**787**	1127	1127	0	1115,2	100,0
**788**	1104	1104	0	1087,8	100,0
**789**	1254	1254	0	1242,1	100,0
**790**	844	843	0	828,3	99,9
**791**	999	999	0	983,6	100,0
**792**	998	998	0	991,6	100,0
**793**	1170	1170	0	1163,4	100,0
**794**	1299	1299	0	1282,8	100,0
**795**	1460	1460	0	1455,2	100,0
**796**	983	983	0	975,6	100,0
**797**	205	205	0	187,3	100,0
**798**	1136	1136	0	1121,4	100,0
**799**	960	960	0	946,1	100,0
**800**	463	463	0	460,3	100,0
**801**	802	802	0	797,1	100,0
**802**	1376	1376	0	1367,4	100,0
**803**	1431	1431	0	1422,8	100,0
**804**	1203	1203	0	1197,2	100,0
**805**	1161	1161	0	1145,4	100,0
**806**	1368	1368	0	1360,3	100,0
**807**	1159	1159	0	1153,4	100,0
**808**	917	917	0	903,7	100,0
**809**	266	266	0	254,8	100,0
**810**	779	779	0	774,4	100,0
**811**	575	575	0	568,8	100,0
**812**	1054	1054	0	1041,7	100,0
**813**	882	882	0	872,7	100,0
**814**	1099	1099	0	1092,4	100,0
**815**	958	958	0	955,5	100,0
**816**	257	257	0	256,0	100,0
**817**	313	313	0	310,4	100,0
**818**	269	269	0	266,9	100,0
**819**	141	141	0	133,8	100,0
**820**	218	218	0	213,8	100,0
**821**	248	248	0	246,0	100,0
**822**	233	233	0	228,9	100,0
**823**	277	277	0	271,3	100,0
**824**	205	205	0	199,6	100,0
**825**	305	305	0	304,4	100,0
**826**	255	255	0	253,1	100,0
**827**	274	274	0	272,1	100,0
**828**	295	295	0	294,5	100,0
**829**	241	241	0	239,2	100,0
**830**	284	284	0	277,6	100,0
**831**	300	300	0	284,7	100,0
**832**	238	238	0	231,2	100,0
**833**	228	228	0	225,4	100,0
**834**	387	387	0	382,1	100,0
**835**	293	293	0	290,7	100,0

**836**	170	170	0	166,8	100,0
**837**	362	362	0	359,3	100,0
**838**	289	289	0	284,1	100,0
**839**	234	234	0	230,9	100,0
**840**	410	410	0	405,7	100,0
**841**	371	371	0	364,5	100,0
**842**	246	246	0	235,3	100,0
**843**	214	214	0	208,3	100,0
**844**	169	169	0	164,9	100,0
**845**	243	243	0	239,3	100,0
**846**	200	200	0	196,4	100,0
**847**	240	240	0	235,0	100,0
**848**	321	321	0	318,1	100,0
**849**	215	215	0	213,7	100,0
**850**	251	251	0	240,1	100,0
**851**	254	254	0	246,0	100,0
**852**	368	368	0	347,8	100,0
**853**	258	258	0	241,2	100,0
**854**	242	242	0	227,4	100,0
**855**	256	256	0	247,8	100,0
**856**	214	214	0	195,7	100,0
**857**	291	291	0	272,5	100,0
**858**	199	199	0	189,1	100,0
**859**	327	327	0	315,9	100,0
**860**	350	350	0	333,6	100,0
**861**	443	443	0	437,0	100,0
**862**	347	347	0	341,2	100,0
**863**	465	465	0	461,9	100,0
**864**	511	509	0	480,1	99,6
**865**	425	425	0	416,9	100,0
**866**	522	522	0	518,2	100,0
**867**	505	505	0	490,4	100,0
**868**	561	561	0	546,5	100,0
**869**	490	490	0	482,2	100,0
**870**	827	827	0	819,5	100,0
**871**	531	531	0	523,6	100,0
**872**	520	520	0	513,6	100,0
**873**	445	445	0	441,5	100,0
**874**	588	581	0	576,7	98,8
**875**	458	458	0	446,1	100,0
**876**	445	445	0	439,5	100,0
**877**	693	693	0	685,7	100,0
**878**	409	409	0	405,8	100,0
**879**	717	717	0	712,6	100,0
**880**	550	550	0	544,3	100,0
**881**	443	443	0	438,1	100,0
**882**	576	576	0	566,8	100,0
**883**	635	633	0	620,3	99,7
**884**	406	406	0	396,1	100,0
**885**	725	725	0	715,7	100,0
**886**	431	431	0	426,4	100,0
**887**	524	524	0	519,3	100,0
**888**	504	504	0	498,0	100,0
**889**	398	398	0	392,3	100,0
**890**	505	505	0	498,1	100,0
**891**	531	531	0	521,8	100,0
**892**	506	506	0	499,8	100,0
**893**	387	387	0	375,6	100,0
**894**	377	377	0	369,7	100,0
**895**	386	386	0	376,2	100,0
**896**	390	390	0	384,7	100,0
**897**	658	641	0	640,2	97,4
**898**	775	775	0	765,8	100,0
**899**	817	817	0	812,5	100,0
**900**	136	136	0	133,4	100,0
**901**	796	794	0	782,0	99,7
**902**	725	725	0	721,4	100,0
**903**	652	652	0	647,4	100,0
**904**	665	665	0	664,4	100,0
**905**	623	623	0	622,4	100,0
**906**	810	810	0	809,6	100,0
**907**	694	693	0	689,9	99,9
**908**	530	530	0	519,0	100,0
**909**	697	697	0	685,9	100,0
**910**	700	700	0	697,2	100,0
**911**	552	552	0	549,9	100,0
**912**	675	675	0	666,1	100,0
**913**	566	566	0	557,8	100,0
**914**	551	551	0	547,1	100,0
**915**	893	893	0	886,3	100,0
**916**	759	758	0	755,3	99,9
**917**	344	344	0	342,9	100,0
**918**	1263	1263	0	1258,1	100,0
**919**	441	441	0	439,3	100,0
**920**	385	338	0	333,6	87,8
**921**	120	120	0	106,7	100,0
**922**	658	658	0	655,2	100,0
**923**	628	628	0	622,6	100,0
**924**	683	682	0	673,2	99,9
**925**	576	576	0	573,8	100,0
**926**	1008	1007	0	996,4	99,9
**927**	691	691	0	686,0	100,0
**928**	685	685	0	675,6	100,0
**929**	721	721	0	718,0	100,0
**930**	536	536	0	523,4	100,0
**931**	718	714	0	696,7	99,4
**932**	682	682	0	666,5	100,0
**933**	751	751	0	725,6	100,0
**934**	609	609	0	598,6	100,0
**935**	714	714	0	695,1	100,0
**936**	728	728	0	705,1	100,0
**937**	512	512	0	497,4	100,0
**938**	758	758	0	735,5	100,0
**939**	795	795	0	775,9	100,0
**940**	913	913	0	898,0	100,0

**941**	521	521	0	503,4	100,0
**942**	677	677	0	673,1	100,0
**943**	880	878	0	866,3	99,8
**944**	790	790	0	775,0	100,0
**945**	770	770	0	765,3	100,0
**946**	360	360	0	352,6	100,0
**947**	960	960	0	938,8	100,0
**948**	964	964	0	952,3	100,0
**949**	765	765	0	749,0	100,0
**950**	774	774	0	764,9	100,0
**951**	831	831	0	823,8	100,0
**952**	882	882	0	877,7	100,0
**953**	653	653	0	643,7	100,0
**954**	659	659	0	632,3	100,0
**955**	645	631	0	627,1	97,8
**956**	758	758	0	743,6	100,0
**957**	755	755	0	752,4	100,0
**958**	637	637	0	629,4	100,0
**959**	677	677	0	659,4	100,0
**960**	469	469	0	460,7	100,0
**961**	604	604	0	596,8	100,0
**962**	603	603	0	594,6	100,0
**963**	672	672	0	669,3	100,0
**964**	368	368	0	364,1	100,0
**965**	886	886	0	883,5	100,0
**966**	547	547	0	527,4	100,0
**967**	593	593	0	587,9	100,0
**968**	721	721	0	702,7	100,0
**969**	808	808	0	796,7	100,0
**970**	704	703	0	694,6	99,9
**971**	656	656	0	636,7	100,0
**972**	747	747	0	740,9	100,0
**973**	509	509	0	501,8	100,0
**974**	837	837	0	824,0	100,0
**975**	751	751	0	748,3	100,0
**976**	586	586	0	583,2	100,0
**977**	842	842	0	833,8	100,0
**978**	495	494	0	485,9	99,8
**979**	642	642	0	633,8	100,0
**980**	534	534	0	515,4	100,0
**981**	525	525	0	522,2	100,0
**982**	514	514	0	506,9	100,0
**983**	565	565	0	542,3	100,0
**984**	623	623	0	614,1	100,0
**985**	455	455	0	444,9	100,0
**986**	547	547	0	539,5	100,0
**987**	532	532	0	526,7	100,0
**988**	590	590	0	585,4	100,0
**989**	861	861	0	858,6	100,0
**990**	654	653	0	651,7	99,8
**991**	208	208	0	197,2	100,0
**992**	461	461	0	459,8	100,0
**993**	658	658	0	649,4	100,0
**994**	559	559	0	557,2	100,0
**995**	586	586	0	583,0	100,0
**996**	753	753	0	752,0	100,0
**997**	796	795	0	790,2	99,9
**998**	802	802	0	798,9	100,0
**999**	757	757	0	754,5	100,0
**1000**	822	822	0	819,5	100,0
**1001**	618	618	0	615,0	100,0
**1002**	565	565	0	555,2	100,0
**1003**	715	715	0	704,9	100,0
**1004**	491	490	0	479,9	99,8
**1005**	624	624	0	620,2	100,0
**1006**	550	550	0	547,1	100,0
**1007**	537	537	0	534,8	100,0
**1008**	491	491	0	488,2	100,0
**1009**	753	753	0	743,1	100,0
**1010**	795	795	0	792,3	100,0
**1011**	601	601	0	591,8	100,0
**1012**	291	291	0	286,1	100,0
**1013**	645	645	0	640,4	100,0
**1014**	456	456	0	446,8	100,0
**1015**	571	571	0	563,6	100,0
**1016**	558	558	0	548,9	100,0
**1017**	541	541	0	537,9	100,0
**1018**	420	420	0	416,6	100,0
**1019**	575	575	0	571,4	100,0
**1020**	729	729	0	722,1	100,0
**1021**	534	534	0	514,5	100,0
**1022**	787	787	0	755,7	100,0
**1023**	758	758	0	743,4	100,0
**1024**	396	396	0	380,6	100,0
**1025**	497	497	0	489,7	100,0
**1026**	639	639	0	620,9	100,0
**1027**	593	593	0	579,4	100,0
**1028**	400	400	0	390,6	100,0
**1029**	599	599	0	582,1	100,0
**1030**	861	861	0	840,8	100,0
**1031**	629	629	0	612,9	100,0
**1032**	379	379	0	376,9	100,0
**1033**	642	642	0	630,0	100,0
**1034**	1000	1000	0	991,8	100,0
**1035**	694	694	0	690,9	100,0
**1036**	686	686	0	670,8	100,0
**1037**	925	925	0	907,6	100,0
**1038**	683	683	0	677,0	100,0
**1039**	556	556	0	550,4	100,0
**1040**	737	737	0	722,7	100,0
**1041**	1076	1076	0	1070,1	100,0
**1042**	1085	1085	0	1076,9	100,0
**1043**	1074	1074	0	1058,1	100,0
**1044**	618	618	0	609,3	100,0
**1045**	1112	1112	0	1104,7	100,0

**1046**	703	703	0	701,8	100,0
**1047**	335	335	0	324,7	100,0
**1048**	453	453	0	449,6	100,0
**1049**	554	554	0	543,7	100,0
**1050**	588	587	0	584,0	99,8
**1051**	424	424	0	420,3	100,0
**1052**	606	606	0	597,9	100,0
**1053**	511	511	0	505,0	100,0
**1054**	632	632	0	628,6	100,0
**1055**	615	615	0	608,3	100,0
**1056**	863	863	0	851,8	100,0
**1057**	887	887	0	878,8	100,0
**1058**	615	615	0	607,6	100,0
**1059**	565	565	0	549,3	100,0
**1060**	587	587	0	581,4	100,0
**1061**	717	717	0	693,9	100,0
**1062**	605	605	0	600,2	100,0
**1063**	458	458	0	453,0	100,0
**1064**	705	705	0	701,6	100,0
**1065**	624	624	0	619,4	100,0
**1066**	776	776	0	767,8	100,0
**1067**	910	910	0	905,9	100,0
**1068**	652	652	0	638,4	100,0
**1069**	716	716	0	709,0	100,0
**1070**	664	664	0	660,2	100,0
**1071**	488	488	0	477,1	100,0
**1072**	526	526	0	520,6	100,0
**1073**	509	509	0	497,5	100,0
**1074**	481	481	0	474,0	100,0
**1075**	313	313	0	309,9	100,0
**1076**	476	476	0	473,6	100,0
**1077**	207	207	0	196,4	100,0
**1078**	703	703	0	701,1	100,0
**1079**	657	657	0	657,0	100,0
**1080**	900	900	0	893,8	100,0
**1081**	657	657	0	652,9	100,0
**1082**	703	703	0	701,6	100,0
**1083**	777	777	0	776,4	100,0
**1084**	838	838	0	834,2	100,0
**1085**	752	752	0	745,5	100,0
**1086**	868	868	0	864,4	100,0
**1087**	786	786	0	779,2	100,0
**1088**	841	841	0	840,2	100,0
**1089**	711	711	0	699,6	100,0
**1090**	614	613	0	610,9	99,8
**1091**	521	521	0	520,0	100,0
**1092**	991	991	0	981,9	100,0
**1093**	709	709	0	701,8	100,0
**1094**	760	760	0	759,2	100,0
**1095**	509	509	0	507,9	100,0
**1096**	736	736	0	732,3	100,0
**1097**	710	710	0	702,8	100,0
**1098**	774	773	0	761,8	99,9
**1099**	931	931	0	923,6	100,0
**1100**	797	797	0	794,1	100,0
**1101**	672	672	0	660,9	100,0
**1102**	637	637	0	635,0	100,0
**1103**	630	630	0	626,9	100,0
**1104**	673	673	0	668,6	100,0
**1105**	757	757	0	731,9	100,0
**1106**	722	722	0	715,3	100,0
**1107**	416	416	0	408,9	100,0
**1108**	824	824	0	800,1	100,0
**1109**	624	624	0	604,3	100,0
**1110**	637	637	0	624,7	100,0
**1111**	661	661	0	651,1	100,0
**1112**	630	630	0	616,5	100,0
**1113**	582	582	0	575,4	100,0
**1114**	1024	1024	0	1017,6	100,0
**1115**	1056	1056	0	1034,1	100,0
**1116**	970	970	0	964,1	100,0
**1117**	609	608	0	584,6	99,8
**1118**	896	896	0	889,0	100,0
**1119**	1079	1079	0	1070,1	100,0
**1120**	934	934	0	918,2	100,0
**1121**	906	906	0	904,4	100,0
**1122**	932	932	0	925,1	100,0
**1123**	1147	1147	0	1140,1	100,0
**1124**	1015	1015	0	1001,3	100,0
**1125**	782	782	0	764,6	100,0
**1126**	583	583	0	569,5	100,0
**1127**	930	930	0	919,9	100,0
**1128**	1046	1046	0	1026,7	100,0
**1129**	792	792	0	791,0	100,0
**1130**	625	625	0	621,6	100,0
**1131**	1181	1181	0	1176,0	100,0
**1132**	986	986	0	980,9	100,0
**1133**	670	670	0	667,8	100,0
**1134**	384	384	0	381,0	100,0
**1135**	855	855	0	845,5	100,0
**1136**	807	807	0	799,3	100,0
**1137**	824	824	0	813,0	100,0
**1138**	805	805	0	790,3	100,0
**1139**	801	801	0	796,5	100,0
**1140**	795	795	0	775,7	100,0
**1141**	727	727	0	722,3	100,0
**1142**	697	697	0	694,6	100,0
**1143**	1026	1026	0	1012,5	100,0
**1144**	679	679	0	669,0	100,0
**1145**	615	615	0	608,3	100,0
**1146**	581	581	0	577,6	100,0
**1147**	846	846	0	835,9	100,0
**1148**	863	863	0	847,3	100,0
**1149**	646	646	0	636,8	100,0
**1150**	541	541	0	534,9	100,0

**1151**	613	613	0	602,9	100,0
**1152**	582	582	0	570,2	100,0
**1153**	616	616	0	612,2	100,0
**1154**	482	482	0	479,6	100,0
**1155**	592	592	0	584,2	100,0
**1156**	593	593	0	581,9	100,0
**1157**	660	660	0	653,2	100,0
**1158**	860	860	0	854,7	100,0
**1159**	624	624	0	622,7	100,0
**1160**	827	827	0	824,5	100,0
**1161**	334	333	0	317,6	99,7
**1162**	807	807	0	802,5	100,0
**1163**	576	576	0	569,3	100,0
**1164**	728	728	0	724,4	100,0
**1165**	653	653	0	644,6	100,0
**1166**	426	426	0	423,6	100,0
**1167**	515	515	0	514,5	100,0
**1168**	570	570	0	564,9	100,0
**1169**	755	755	0	748,8	100,0
**1170**	667	667	0	652,4	100,0
**1171**	723	723	0	707,6	100,0
**1172**	737	737	0	724,8	100,0
**1173**	724	723	0	687,8	99,9
**1174**	539	539	0	521,5	100,0
**1175**	643	643	0	636,0	100,0
**1176**	545	545	0	531,1	100,0
**1177**	659	659	0	636,1	100,0
**1178**	620	620	0	605,0	100,0
**1179**	376	376	0	358,5	100,0
**1180**	576	576	0	561,8	100,0
**1181**	657	657	0	648,4	100,0
**1182**	656	656	0	652,1	100,0
**1183**	341	341	0	337,7	100,0
**1184**	727	727	0	721,1	100,0
**1185**	617	617	0	612,1	100,0
**1186**	688	688	0	682,6	100,0
**1187**	619	619	0	609,0	100,0
**1188**	890	890	0	882,8	100,0
**1189**	863	863	0	856,0	100,0
**1190**	543	543	0	540,7	100,0
**1191**	581	581	0	576,2	100,0
**1192**	776	776	0	768,3	100,0
**1193**	576	576	0	572,5	100,0
**1194**	485	485	0	480,5	100,0
**1195**	443	441	0	437,3	99,5
**1196**	409	409	0	404,3	100,0
**1197**	513	513	0	508,6	100,0
**1198**	418	418	0	414,4	100,0
**1199**	490	490	0	483,8	100,0
**1200**	387	387	0	381,0	100,0
**1201**	603	602	0	598,6	99,8
**1202**	375	375	0	372,2	100,0
**1203**	426	426	0	425,1	100,0
**1204**	801	801	0	797,7	100,0
**1205**	594	594	0	591,5	100,0
**1206**	816	816	0	813,6	100,0
**1207**	307	306	0	294,2	99,7
**1208**	543	543	0	536,6	100,0
**1209**	719	719	0	714,6	100,0
**1210**	441	441	0	438,9	100,0
**1211**	657	657	0	654,8	100,0
**1212**	463	463	0	459,7	100,0
**1213**	618	618	0	615,0	100,0
**1214**	496	496	0	493,5	100,0
**1215**	664	664	0	660,8	100,0
**1216**	643	642	0	637,7	99,8
**1217**	648	648	0	641,8	100,0
**1218**	708	708	0	703,1	100,0
**1219**	832	832	0	813,3	100,0
**1220**	607	607	0	603,9	100,0
**1221**	622	622	0	613,8	100,0
**1222**	565	565	0	560,5	100,0
**1223**	622	622	0	615,3	100,0
**1224**	556	556	0	547,8	100,0
**1225**	396	396	0	392,6	100,0
**1226**	666	666	0	661,3	100,0
**1227**	645	645	0	638,0	100,0
**1228**	810	810	0	804,3	100,0
**1229**	614	614	0	608,9	100,0
**1230**	791	791	0	787,1	100,0
**1231**	744	744	0	725,3	100,0
**1232**	612	612	0	604,0	100,0
**1233**	773	773	0	766,4	100,0
**1234**	688	687	0	680,2	99,9
**1235**	825	825	0	814,0	100,0
**1236**	593	593	0	583,9	100,0
**1237**	493	493	0	484,4	100,0
**1238**	567	567	0	561,4	100,0
**1239**	805	805	0	798,6	100,0
**1240**	450	449	0	447,6	99,8
**1241**	506	505	0	500,7	99,8
**1242**	577	577	0	570,7	100,0
**1243**	635	633	0	624,3	99,7
**1244**	595	595	0	590,5	100,0
**1245**	754	754	0	747,7	100,0
**1246**	564	564	0	562,6	100,0
**1247**	557	557	0	549,0	100,0
**1248**	437	437	0	432,4	100,0
**1249**	761	761	0	756,5	100,0
**1250**	692	692	0	687,2	100,0
**1251**	370	370	0	363,7	100,0
**1252**	796	796	0	779,8	100,0
**1253**	834	834	0	827,6	100,0
**1254**	611	611	0	595,6	100,0
**1255**	1003	1003	0	1000,4	100,0

**1256**	511	511	0	508,6	100,0
**1257**	860	860	0	856,3	100,0
**1258**	804	803	0	799,4	99,9
**1259**	770	770	0	755,2	100,0
**1260**	818	818	0	781,7	100,0
**1261**	705	704	0	687,6	99,9
**1262**	635	634	0	616,4	99,8
**1263**	608	608	0	562,8	100,0
**1264**	641	641	0	626,5	100,0
**1265**	624	624	0	612,4	100,0
**1266**	765	765	0	742,8	100,0
**1267**	816	816	0	799,7	100,0
**1268**	368	368	0	352,7	100,0
**1269**	666	665	0	639,3	99,8
**1270**	495	495	0	463,9	100,0
**1271**	855	848	0	841,7	99,2
**1272**	2207	2207	0	2183,9	100,0
**1273**	555	555	0	548,6	100,0
**1274**	849	849	0	841,5	100,0
**1275**	746	746	0	729,8	100,0
**1276**	913	913	0	907,7	100,0
**1277**	659	659	0	653,5	100,0
**1278**	1333	1333	0	1320,7	100,0
**1279**	723	723	0	718,4	100,0
**1280**	729	729	0	727,9	100,0
**1281**	1007	1007	0	998,3	100,0
**1282**	556	555	0	543,6	99,8
**1283**	572	572	0	565,5	100,0
**1284**	633	633	0	619,5	100,0
**1285**	577	576	0	570,6	99,8
**1286**	448	448	0	443,5	100,0
**1287**	626	626	0	618,4	100,0
**1288**	474	474	0	473,1	100,0
**1289**	625	624	0	618,8	99,8
**1290**	521	521	0	519,9	100,0
**1291**	615	615	0	611,0	100,0
**1292**	503	503	0	497,9	100,0
**1293**	541	516	0	508,6	95,4
**1294**	798	798	0	795,0	100,0
**1295**	738	738	0	718,2	100,0
**1296**	533	533	0	527,4	100,0
**1297**	200	200	0	195,6	100,0
**1298**	552	552	0	544,3	100,0
**1299**	734	734	0	725,1	100,0
**1300**	582	582	0	577,8	100,0
**1301**	816	816	0	806,0	100,0
**1302**	726	726	0	725,6	100,0
**1303**	497	497	0	493,0	100,0
**1304**	744	744	0	739,8	100,0
**1305**	562	562	0	551,2	100,0
**1306**	777	776	0	768,3	99,9
**1307**	849	849	0	834,3	100,0
**1308**	740	740	0	734,5	100,0
**1309**	795	795	0	786,3	100,0
**1310**	570	570	0	567,4	100,0
**1311**	669	669	0	668,7	100,0
**1312**	991	991	0	982,7	100,0
**1313**	787	786	0	781,5	99,9
**1314**	422	422	0	416,3	100,0
**1315**	821	821	0	814,6	100,0
**1316**	527	527	0	499,2	100,0
**1317**	866	866	0	852,5	100,0
**1318**	826	826	0	821,9	100,0
**1319**	803	803	0	796,7	100,0
**1320**	824	824	0	804,6	100,0
**1321**	1030	1030	0	1022,6	100,0
**1322**	874	874	0	871,1	100,0
**1323**	1062	1062	0	1053,7	100,0
**1324**	692	691	0	683,9	99,9
**1325**	777	777	0	768,9	100,0
**1326**	948	948	0	939,4	100,0
**1327**	555	555	0	541,9	100,0
**1328**	811	811	0	808,6	100,0
**1329**	679	679	0	677,2	100,0
**1330**	718	718	0	712,8	100,0
**1331**	811	811	0	802,2	100,0
**1332**	705	705	0	700,8	100,0
**1333**	606	606	0	604,9	100,0
**1334**	387	387	0	379,9	100,0
**1335**	695	695	0	693,7	100,0
**1336**	724	724	0	718,3	100,0
**1337**	641	641	0	634,0	100,0
**1338**	573	573	0	571,5	100,0
**1339**	283	283	0	276,0	100,0
**1340**	942	942	0	935,1	100,0
**1341**	464	464	0	456,1	100,0
**1342**	419	419	0	414,6	100,0
**1343**	507	506	0	500,0	99,8
**1344**	471	471	0	468,3	100,0
**1345**	664	664	0	658,3	100,0
**1346**	996	996	0	993,3	100,0
**1347**	651	651	0	644,7	100,0
**1348**	757	757	0	743,7	100,0
**1349**	684	684	0	671,8	100,0
**1350**	458	458	0	431,0	100,0
**1351**	583	583	0	558,2	100,0
**1352**	472	472	0	442,4	100,0
**1353**	479	479	0	460,9	100,0
**1354**	615	615	0	595,6	100,0
**1355**	512	511	0	491,7	99,8
**1356**	338	338	0	328,1	100,0
**1357**	639	639	0	622,0	100,0
**1358**	686	686	0	664,2	100,0
**1359**	1227	1227	0	1215,5	100,0
**1360**	1167	1167	0	1162,6	100,0

**1361**	768	768	0	762,6	100,0
**1362**	568	568	0	556,6	100,0
**1363**	1090	1090	0	1085,9	100,0
**1364**	659	659	0	639,2	100,0
**1365**	558	558	0	556,4	100,0
**1366**	1044	1044	0	1037,1	100,0
**1367**	570	570	0	564,7	100,0
**1368**	747	747	0	741,3	100,0
**1369**	779	778	0	774,6	99,9
**1370**	537	536	0	528,6	99,8
**1371**	567	566	0	560,1	99,8
**1372**	427	427	0	423,1	100,0
**1373**	382	382	0	373,2	100,0
**1374**	742	742	0	740,4	100,0
**1375**	497	497	0	483,8	100,0
**1376**	442	442	0	433,6	100,0
**1377**	407	407	0	401,5	100,0
**1378**	732	731	0	720,8	99,9
**1379**	648	648	0	645,6	100,0
**1380**	687	687	0	681,8	100,0
**1381**	433	433	0	427,5	100,0
**1382**	574	573	0	564,6	99,8
**1383**	676	676	0	669,6	100,0
**1384**	571	571	0	564,5	100,0
**1385**	192	191	0	176,7	99,5
**1386**	877	877	0	871,4	100,0
**1387**	462	462	0	444,9	100,0
**1388**	501	501	0	495,2	100,0
**1389**	552	552	0	548,5	100,0
**1390**	441	441	0	438,6	100,0
**1391**	621	621	0	613,6	100,0
**1392**	1137	1137	0	1130,7	100,0
**1393**	809	809	0	806,2	100,0
**1394**	356	356	0	348,7	100,0
**1395**	747	746	0	738,6	99,9
**1396**	788	788	0	784,3	100,0
**1397**	622	622	0	609,7	100,0
**1398**	1097	1096	0	1089,3	99,9
**1399**	616	616	0	592,9	100,0
**1400**	518	518	0	515,5	100,0
**1401**	608	608	0	602,2	100,0
**1402**	786	786	0	777,1	100,0
**1403**	344	344	0	338,9	100,0
**1404**	1043	1043	0	1038,0	100,0
**1405**	780	780	0	772,7	100,0
**1406**	659	659	0	651,1	100,0
**1407**	598	598	0	593,9	100,0
**1408**	751	751	0	742,1	100,0
**1409**	490	490	0	475,0	100,0
**1410**	713	713	0	704,9	100,0
**1411**	673	673	0	652,2	100,0
**1412**	550	550	0	544,6	100,0
**1413**	790	790	0	787,4	100,0
**1414**	1041	1041	0	1033,5	100,0
**1415**	569	569	0	564,8	100,0
**1416**	743	743	0	733,3	100,0
**1417**	844	844	0	837,1	100,0
**1418**	646	646	0	641,4	100,0
**1419**	622	622	0	620,5	100,0
**1420**	584	584	0	578,6	100,0
**1421**	451	451	0	437,9	100,0
**1422**	586	586	0	576,9	100,0
**1423**	513	513	0	499,7	100,0
**1424**	427	427	0	423,6	100,0
**1425**	476	475	0	471,4	99,8
**1426**	855	855	0	851,2	100,0
**1427**	997	997	0	991,9	100,0
**1428**	978	976	0	962,9	99,8
**1429**	1065	1065	0	1063,0	100,0
**1430**	482	482	0	470,0	100,0
**1431**	617	616	0	613,7	99,8
**1432**	599	599	0	595,9	100,0
**1433**	837	837	0	829,4	100,0
**1434**	850	850	0	842,0	100,0
**1435**	715	715	0	711,8	100,0
**1436**	565	565	0	560,9	100,0
**1437**	639	639	0	632,6	100,0
**1438**	709	709	0	703,8	100,0
**1439**	1155	1155	0	1149,2	100,0
**1440**	694	694	0	667,1	100,0
**1441**	688	688	0	672,7	100,0
**1442**	634	634	0	606,2	100,0
**1443**	774	774	0	758,3	100,0
**1444**	541	541	0	528,8	100,0
**1445**	1085	1085	0	1068,5	100,0
**1446**	684	684	0	664,1	100,0
**1447**	650	650	0	642,4	100,0
**1448**	398	398	0	389,5	100,0
**1449**	740	740	0	720,2	100,0
**1450**	773	773	0	749,4	100,0
**1451**	1387	1387	0	1382,6	100,0
**1452**	762	762	0	744,8	100,0
**1453**	795	795	0	792,0	100,0
**1454**	620	620	0	617,5	100,0
**1455**	990	990	0	981,1	100,0
**1456**	1132	1132	0	1123,9	100,0
**1457**	822	822	0	818,0	100,0
**1458**	528	528	0	522,2	100,0
**1459**	811	811	0	806,5	100,0
**1460**	602	602	0	593,4	100,0
**1461**	637	637	0	631,0	100,0
**1462**	564	564	0	558,4	100,0
**1463**	664	663	0	655,6	99,8
**1464**	574	573	0	557,1	99,8
**1465**	593	593	0	590,6	100,0

**1466**	771	771	0	763,1	100,0
**1467**	571	571	0	567,6	100,0
**1468**	343	343	0	328,2	100,0
**1469**	674	674	0	668,7	100,0
**1470**	899	899	0	887,4	100,0
**1471**	591	591	0	588,4	100,0
**1472**	817	817	0	808,9	100,0
**1473**	1187	1187	0	1181,0	100,0
**1474**	929	929	0	914,8	100,0
**1475**	620	620	0	602,8	100,0
**1476**	758	758	0	751,1	100,0
**1477**	395	395	0	389,1	100,0
**1478**	87	87	0	82,5	100,0
**1479**	237	236	0	224,6	99,6
**1480**	296	296	0	289,6	100,0
**1481**	5836	5836	0	5779,4	100,0
**1482**	5416	5416	0	5309,4	100,0
**1483**	3553	3551	0	3508,2	99,9
**1484**	2667	2667	0	2611,4	100,0
**1485**	7129	7128	0	7055,5	100,0
**1486**	7365	7365	0	7239,3	100,0
**1487**	3624	3623	0	3572,8	100,0
**1488**	4331	4331	0	4251,5	100,0
**1489**	6227	6226	0	6148,7	100,0
**1490**	6009	6009	0	5867,4	100,0
**1491**	9527	9527	0	9490,5	100,0
**1492**	6460	6460	0	6428,5	100,0
**1493**	5475	5475	0	5355,5	100,0
**1494**	3374	3373	0	3272,9	100,0
**1495**	3603	3603	0	3566,3	100,0
**1496**	10299	10295	0	10226,0	100,0
**1497**	6464	6464	0	6272,7	100,0
**1498**	7236	7236	0	7205,2	100,0
**1499**	6933	6933	0	6822,5	100,0
**1500**	7118	7117	0	7016,2	100,0
**1501**	8044	8044	0	7950,0	100,0
**1502**	3124	3124	0	3022,8	100,0
**1503**	5875	5875	0	5819,1	100,0
**1504**	7859	7859	0	7776,4	100,0
**1505**	6673	6672	0	6597,6	100,0
**1506**	4055	4055	0	4011,4	100,0
**1507**	3433	3433	0	3316,5	100,0
**1508**	5749	5748	0	5692,6	100,0
**1509**	6337	6337	0	6276,0	100,0
**1510**	4366	4366	0	4261,5	100,0
**1511**	2960	2960	0	2917,4	100,0
**1512**	8426	8425	0	8335,6	100,0
**1513**	7482	7479	0	7370,4	100,0
**1514**	4123	4123	0	4052,8	100,0
**1515**	4575	4575	0	4465,2	100,0
**1516**	4834	4833	0	4814,1	100,0
**1517**	5399	5399	0	5389,5	100,0
**1518**	5077	5071	0	5030,4	99,9
**1519**	3877	3876	0	3825,7	100,0
**1520**	5308	5308	0	5280,2	100,0
**1521**	5661	5661	0	5587,4	100,0
**1522**	4222	4221	0	4135,5	100,0
**1523**	4575	4574	0	4529,3	100,0
**1524**	3643	3642	0	3551,7	100,0
**1525**	3273	3273	0	3206,9	100,0
**1526**	4777	4777	0	4692,1	100,0
**1527**	3416	3416	0	3327,6	100,0
**1528**	3663	3661	0	3562,5	99,9
**1529**	2754	2754	0	2681,1	100,0
**1530**	657	657	0	596,7	100,0
**1531**	4884	4884	0	4763,7	100,0
**1532**	2962	2962	0	2886,6	100,0
**1533**	3127	3127	0	3021,2	100,0
**1534**	5657	5655	0	5542,2	100,0
**1535**	5035	5034	0	4843,2	100,0
**1536**	5273	5272	0	5122,6	100,0
**1537**	4088	4088	0	4015,5	100,0
**1538**	5008	5008	0	4897,2	100,0
**1539**	2624	2624	0	2504,0	100,0
**1540**	5239	5239	0	5189,7	100,0
**1541**	4837	4834	0	4745,9	99,9
**1542**	6503	6503	0	6339,7	100,0
**1543**	6613	6612	0	6564,8	100,0
**1544**	2752	2752	0	2625,1	100,0
**1545**	2893	2892	0	2805,7	100,0
**1546**	2054	2053	0	2019,7	100,0
**1547**	1193	1192	0	1138,2	99,9
**1548**	1441	1441	0	1416,3	100,0
**1549**	5267	5267	0	5231,3	100,0
**1550**	1193	1193	0	1169,0	100,0
**1551**	2182	2182	0	2107,3	100,0
**1552**	3464	3464	0	3406,5	100,0
**1553**	1660	1660	0	1617,8	100,0
**1554**	3018	3018	0	2978,2	100,0
**1555**	2433	2432	0	2367,5	100,0
**1556**	1913	1913	0	1888,6	100,0
**1557**	4893	4893	0	4872,6	100,0
**1558**	5043	5041	0	4975,6	100,0
**1559**	7244	7244	0	7152,3	100,0
**1560**	4347	4347	0	4270,9	100,0
**1561**	1890	1890	0	1844,4	100,0
**1562**	5644	5644	0	5531,3	100,0
**1563**	5755	5755	0	5597,3	100,0
**1564**	4772	4771	0	4692,3	100,0
**1565**	6266	6266	0	6174,2	100,0
**1566**	6284	6284	0	6142,9	100,0
**1567**	8511	8511	0	8361,3	100,0
**1568**	6629	6629	0	6487,1	100,0

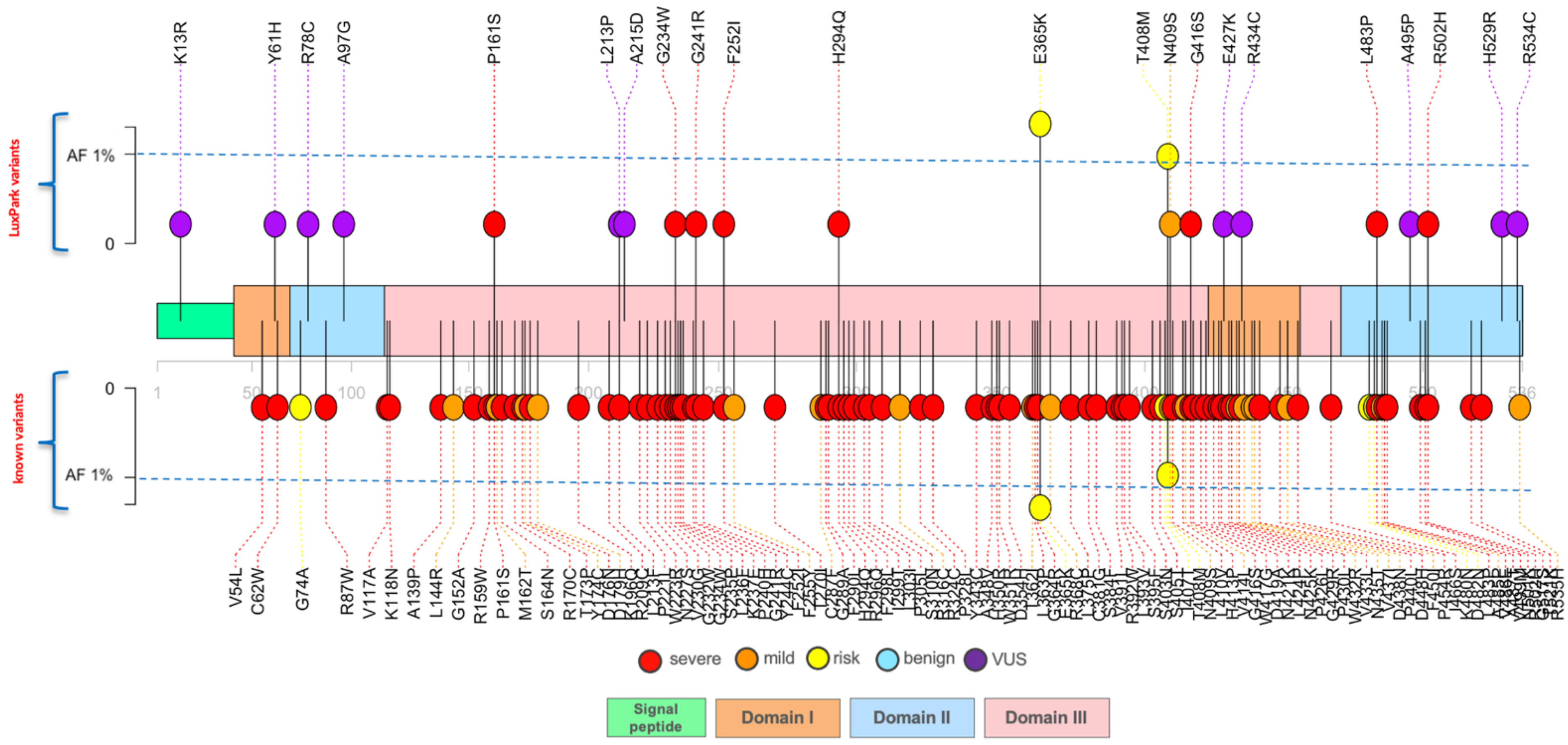

Supplementary Figure 1: Lolliplot graphics showing all *GBA1* variants found in the Luxembourg Parkinson's study through the whole protein sequences. Each lollipop represents a *GBA1* variant identified in this the Luxembourgish cohort (upper part) and known *GBA1* variants in ClinVar or literatures (bottom part). Variants classified as severe are colored red, mild are colored orange, risk in yellow and VUS are colored purple.

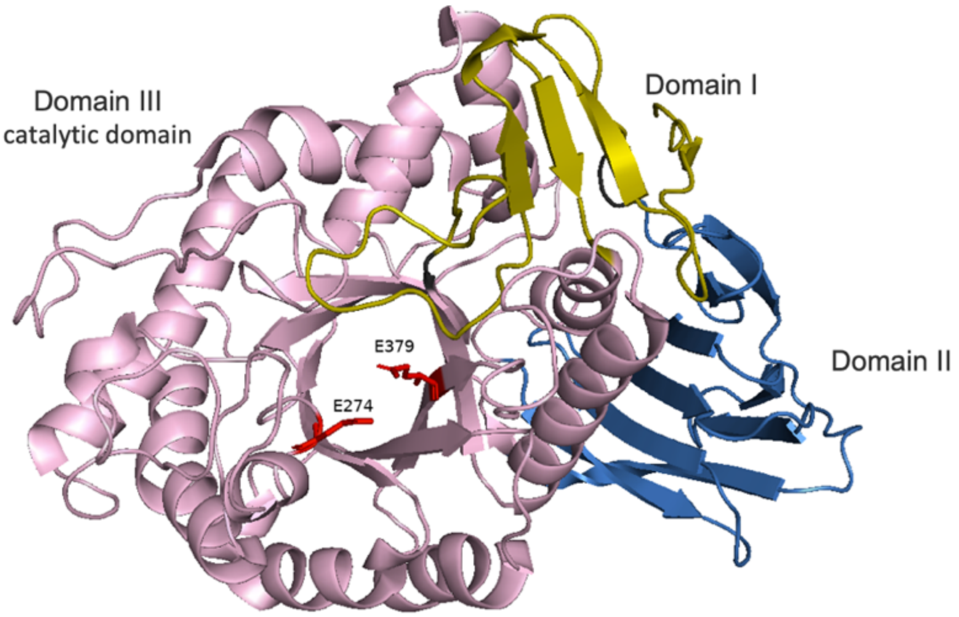

Supplementary Figure 2. The 3D structure of GCase (PDB code 1ogs) created using PYMOL (http://www.pymol.org). Domain I is shown in dark yellow with the antiparallel β sheet (residues 1–27 and 383–414), Domain II in blue with the immunoglobulin-like domain (residues 30–75 and 431–497), and Domain III in pink is the catalytic domain with the (b/a)8 (TIM) barrel structure (residues 76–381 and 416–430). The active site residues Glu274 and Glu379 are shown in red.

Supplementary References

1. Nomura, T., Inoue, Y., Kagimura, T., Uemura, Y. & Nakashima, K. Utility of the REM sleep behavior disorder screening questionnaire (RBDSQ) in Parkinson's disease patients. *Sleep Med.* **12**, 711–713 (2011).
2. Chaudhuri, K. R. *et al.* The Parkinson's disease sleep scale: a new instrument for assessing sleep and nocturnal disability in Parkinson's disease. *J. Neurol. Neurosurg. Psychiatry* **73**, 629–635 (2002).
3. Tomlinson, C. L. *et al.* Systematic review of levodopa dose equivalency reporting in Parkinson's disease. *Mov. Disord. Off. J. Mov. Disord. Soc.* **25**, 2649–2653 (2010).
4. Longstreth, G. F. *et al.* Functional bowel disorders. *Gastroenterology* **130**, 1480–1491 (2006).
5. Goetz, C. G. *et al.* Movement Disorder Society Task Force report on the Hoehn and Yahr staging scale: status and recommendations. *Mov. Disord. Off. J. Mov. Disord. Soc.* **19**, 1020–1028 (2004).
6. Peto, V., Jenkinson, C., Fitzpatrick, R. & Greenhall, R. The development and validation of a short measure of functioning and well being for individuals with Parkinson's disease. *Qual. Life Res. Int. J. Qual. Life Asp. Treat. Care Rehabil.* **4**, 241–248 (1995).
7. Beck, A. T., Ward, C. H., Mendelson, M., Mock, J. & Erbaugh, J. An inventory for measuring depression. *Arch. Gen. Psychiatry* **4**, 561–571 (1961).
8. Hummel, T., Kobal, G., Gudziol, H. & Mackay-Sim, A. Normative data for the 'Sniffin' Sticks' including tests of odor identification, odor discrimination, and olfactory thresholds: an upgrade based on a group of more than 3,000 subjects. *Eur. Arch. Oto-Rhino-Laryngol. Off. J. Eur. Fed. Oto-Rhino-Laryngol. Soc. EUFOS Affil. Ger. Soc. Oto-Rhino-Laryngol. - Head Neck Surg.* **264**, 237–243 (2007).
9. Nasreddine, Z. S. *et al.* The Montreal Cognitive Assessment, MoCA: a brief screening tool for mild cognitive impairment. *J. Am. Geriatr. Soc.* **53**, 695–699 (2005).
10. Ruskey, J. A. *et al.* Increased yield of full GBA sequencing in Ashkenazi Jews with Parkinson's disease. *Eur. J. Med. Genet.* **62**, 65–69 (2019).
11. den Heijer, J. M. *et al.* A Large-Scale Full GBA1 Gene Screening in Parkinson's Disease in the Netherlands. *Mov. Disord. Off. J. Mov. Disord. Soc.* **35**, 1667–1674 (2020).
12. Petrucci, S. *et al.* GBA-Related Parkinson's Disease: Dissection of Genotype-Phenotype Correlates in a Large Italian Cohort. *Mov. Disord. Off. J. Mov. Disord. Soc.* **35**, 2106–2111 (2020).
13. Jesús, S. *et al.* GBA Variants Influence Motor and Non-Motor Features of Parkinson's Disease. *PloS One* **11**, e0167749 (2016).
14. Graham, O. E. E. *et al.* Nanopore sequencing of the glucocerebrosidase (GBA) gene in a New Zealand Parkinson's disease cohort. *Parkinsonism Relat. Disord.* **70**, 36–41 (2020).
15. Olszewska, D. A. *et al.* Association Between Glucocerebrosidase Mutations and Parkinson's Disease in Ireland. *Front. Neurol.* **11**, 527 (2020).
16. Bras, J. *et al.* Complete screening for glucocerebrosidase mutations in Parkinson disease patients from Portugal. *Neurobiol. Aging* **30**, 1515–1517 (2009).
17. Kalinderi, K. *et al.* Complete screening for glucocerebrosidase mutations in Parkinson disease patients from Greece. *Neurosci. Lett.* **452**, 87–89 (2009).
